# Microgliosis, neuronal death, minor behavioral abnormalities and reduced endurance performance in alpha-ketoglutarate dehydrogenase complex deficient mice^☆^

**DOI:** 10.1016/j.redox.2025.103743

**Published:** 2025-06-27

**Authors:** Márton Kokas, András Budai, Andrea Kádár, Soroosh Mozaffaritabar, Lei Zhou, Tímea Téglás, Rebeka Sára Orova, Dániel Gáspár, Kristóf Németh, Daniel Marton Toth, Nabil V. Sayour, Csenger Kovácsházi, Andrea Xue, Réka Zsuzsanna Szatmári, Beáta Törőcsik, Domokos Máthé, Noémi Kovács, Krisztián Szigeti, Péter Nagy, Ildikó Szatmári, Csaba Fekete, Tamás Arányi, Zoltán V. Varga, Péter Ferdinandy, Zsolt Radák, Andrey V. Kozlov, László Tretter, Tímea Komlódi, Attila Ambrus

**Affiliations:** a Department of Biochemistry, Semmelweis University, 37-47 Tuzolto Street, Budapest, 1094, Hungary; b Department of Pathology, Forensic and Insurance Medicine, Semmelweis University, 93 Ulloi Street, Budapest, 1091, Hungary; c Laboratory for Integrative Neuroendocrinology, HUN-REN Institute of Experimental Medicine, 43 Szigony Street, Budapest, 1083, Hungary; d Research Center for Molecular Exercise Science, Hungarian University of Sport Sciences, 44-48 Alkotas Street, Budapest, 1123, Hungary; e Department of Molecular Biology, Semmelweis University, 37-47 Tuzolto Street, Budapest, 1094, Hungary; f Department of Pharmacology and Pharmacotherapy, Semmelweis University, 4 Nagyvarad Square, Budapest, 1089, Hungary; g Center for Pharmacology, Drug Research and Development, Semmelweis University, 4 Nagyvarad Square, Budapest, 1089, Hungary; h MTA-SE Momentum Cardio-Oncology and Cardioimmunology Research Group, Semmelweis University, 4 Nagyvarad Square, Budapest, 1089, Hungary; i Pediatric Centre, MTA Centre of Excellence, Semmelweis University, 53-54 Bokay Janos Street, Budapest, 1083, Hungary; j Department of Molecular Immunology and Toxicology and the National Tumor Biology Laboratory, National Institute of Oncology, 7-9 Rath Gyorgy Street, Budapest, 1122, Hungary; k Laki Kálmán Doctoral School, University of Debrecen, 98 Nagyerdei Boulevard, Debrecen, 4032, Hungary; l Department of Biophysics and Radiation Biology, Semmelweis University, 37-47 Tuzolto Street, Budapest, 1094, Hungary; m Hungarian Centre of Excellence for Molecular Medicine, 9 Budapesti Street, Szeged, 6728, Hungary; n Chemistry Institute, University of Debrecen, 1 Egyetem Square, Debrecen, 4032, Hungary; o Department of Anatomy and Histology, HUN-REN–UVMB Laboratory of Redox Biology Research Group, University of Veterinary Medicine, 2 Istvan Street, Budapest, 1078, Hungary; p HUN-REN Research Centre for Natural Sciences, Institute of Molecular Life Sciences, 2 Magyar Tudosok Boulevard, Budapest, 1117, Hungary; q Pharmahungary Group, 6 Hajnoczy Street, Szeged, 6722, Hungary; r Ludwig Boltzmann Institute, 13 Donaueschingenstrasse, Vienna, 1200, Austria

**Keywords:** Alpha-ketoglutarate dehydrogenase complex, Dihydrolipoyl succinyltransferase, Dihydrolipoyl dehydrogenase, Cognitive decline, Reactive oxygen species, Fatigue test

## Abstract

The alpha-ketoglutarate dehydrogenase complex (KGDHc), also known as the 2-oxoglutarate dehydrogenase complex, plays a crucial role in oxidative metabolism. It catalyzes a key step in the tricarboxylic acid (TCA) cycle, producing NADH (primarily for oxidative phosphorylation) and succinyl-CoA (for substrate-level phosphorylation, among others). Additionally, KGDHc is also capable of generating reactive oxygen species, which contribute to mitochondrial oxidative stress. Hence, the KGDHc and its dysfunction are implicated in various pathological conditions, including selected neurodegenerative diseases. The pathological roles of KGDHc in these diseases are generally still obscure.

The aim of this study was to assess whether the mitochondrial malfunctions observed in the dihydrolipoamide succinyltransferase (*DLST*) and dihydrolipoamide dehydrogenase (*DLD*) double-heterozygous knockout (DLST^+/−^DLD^+/−^, DKO) mice are associated with neuronal and/or metabolic abnormalities.

In the DKO animals, the mitochondrial O_2_ consumption and ATP production rates both decreased in a substrate-specific manner. Reduced H_2_O_2_ production was also observed, either due to Complex I inhibition with α-ketoglutarate or reverse electron transfer with succinate, which is significant in ischaemia-reperfusion injury. Middle-aged DKO mice exhibited minor cognitive decline, associated with microgliosis in the cerebral cortex and neuronal death in the *Cornu Ammonis* subfield 1 (CA1) of the hippocampus, indicating neuroinflammation. This was supported by increased levels of dynamin-related protein 1 (Drp1) and reduced levels of mitofusin 2 and peroxisome proliferator-activated receptor gamma coactivator 1-alpha (PGC-1α) in DKO mice. Observations on activity, food and oxygen consumption, and blood amino acid and acylcarnitine profiles revealed no significant differences. However, middle-aged DKO animals showed decreased performance in the treadmill fatigue-endurance test as compared to wild-type animals, accompanied by subtle resting cardiac impairment, but not skeletal muscle fibrosis.

In conclusion, DKO animals compensate well the double-heterozygous knockout condition at the whole-body level with no major phenotypic changes under resting physiological conditions. However, under high energy demand, middle-aged DKO mice exhibited reduced performance, suggesting a decline in metabolic compensation. Additionally, microgliosis, neuronal death, decreased mitochondrial biogenesis, and altered mitochondrial dynamics were observed in DKO animals, resulting in minor cognitive decline. This is the first study to highlight the *in vivo* changes of this combined genetic modification. It demonstrates that unlike single knockout rodents, double knockout mice exhibit phenotypical alterations that worsen under stress situations.

## Highlights

• Reduced H_2_O_2_ efflux with succinate or α-ketoglutarate in mutant mitochondria
• Decreased performance of the mutant animals in the fatigue-endurance test
• Microgliosis, neuronal death and mild cognitive decline in the mutant mice
• Altered mitochondrial dynamics and biogenesis in the deficient mice

## Graphical abstract

**Figure:**
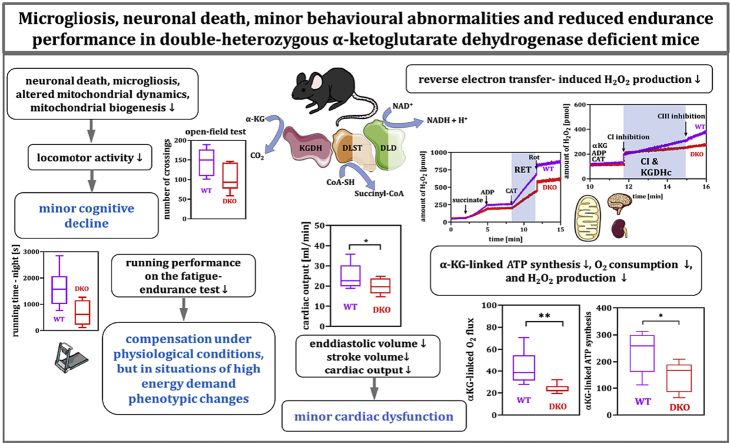

## Abbreviations

ΔΨ_m_: mitochondrial membrane potential
AD: Alzheimer's disease
ADP: adenosine diphosphate
α-KG: alpha-ketoglutarate
KGDH: alpha-ketoglutarate dehydrogenase (E1 subunit)
KGDHc: alpha-ketoglutarate dehydrogenase complex
Ama: antimycin A
ANT: adenine nucleotide translocase
AP5: P^1^,P^5^-di(adenosine-5′-)pentaphosphate
ATP: adenosine triphosphate
BCKDHc: branched-chain alpha-keto acid dehydrogenase complex
CI: Complex I
CII: Complex II
CIII: Complex III
CIV: Complex IV
CA1: *Cornu Ammonis* subfield 1
CAT: carboxyatractylozide;
CD68: cluster of differentiation 68
CoA-SH: coenzyme A reduced form
DAB: 3,3′-diaminobenzidine;
DBS: dried blood spots
*DLD*: gene encoding dihydrolipoamide dehydrogenase (E3 subunit)
*DLST*: gene encoding dihydrolipoyl succinyltransferase (E2 subunit)
DKO: double heterozygous knock-out for *DLST* and *DLD*
Drp1: dynamin-related protein 1
EDV: end diastolic volume
EF: ejection fraction
EGTA: ethylene glycol-bis(β-aminoethyl ether)-N,N,N′,N'-tetraacetic acid
ESV: end systolic volume
ETS: electron transfer system
FCCP: carbonyl cyanide-*p*-trifluoromethoxyphenylhydrazone
FDG: F-18-fluorodeoxyglucose
FS: fractional shortening
GCS: glycine cleavage system
G6PDH: glucose-6-phosphate dehydrogenase
GTP: guanosine triphosphate
HEPES: 4-(2-hydroxyethyl)-1-piperazineethanesulfonic acid
HK: hexokinase
IVRT: isovolumetric relaxation time;
KADHc: alpha-ketoadipate dehydrogenase complex
LV: left ventricular
LVAWd: end diastolic LV anterior wall thicknesses
LVAWs: end systolic LV anterior wall thicknesses
LVIDd: end diastolic left ventricular diameter
LVIDs: end systolic left ventricular diameter
LVPWd: end diastolic LV posterior wall thicknesses
LVPWs: end systolic LV posterior wall thicknesses
Mfn2: mitofusin 2
MTFA: mitochondrial transcription factor A
NADH: nicotinamide adenine dinucleotide (reduced)
NADPH: nicotinamide adenine dinucleotide phosphate (reduced)
Nrf2: nuclear factor (erythroid-derived 2)-like 2
OXPHOS: oxidative phosphorylation
PCR: polymerase chain reaction
PD: Parkinson's disease
PDHc: pyruvate dehydrogenase complex
PET: positron emission tomography
PGC-1α: peroxisome proliferator-activated receptor gamma coactivator 1-alpha
PMSF: phenylmethyl sulfonyl fluoride;
POS: polarographic oxygen sensor
RER: respiratory exchange ratio
RET: reverse electron transfer
ROS: reactive oxygen species
Rot: rotenone
Rps29: ribosomal protein S29
SDS: sodium dodecyl sulphate
SLP: substrate-level phosphorylation
SUV: standardized uptake values
SV: stroke volume
TBST: Tris-buffered saline with Tween-20
TCA cycle: tricarboxylic acid cycle
TUNEL: terminal deoxynucleotidyl transferase mediated dUTP-biotin nick end labelling

## 1 Introduction

The alpha-ketoglutarate dehydrogenase complex (KGDHc), also known as 2-oxoglutarate dehydrogenase complex, plays a crucial role in the tricarboxylic acid (TCA) cycle by catalyzing the oxidative decarboxylation of α-ketoglutarate (α-KG) to succinyl-CoA (α-KG + NAD^+^ + CoA-SH → succinyl-CoA + NADH + H^+^ + CO_2_; EC1.2.4.2) [1]. The KGDHc is a multienzyme complex consisting of three types of subunits: α-ketoglutarate dehydrogenase (*KGDH/OGDH*, E1k; EC 1.8.1.4), dihydrolipoyl succinyltransferase (*DLST*, E2k; EC 2.3.1.61), and dihydrolipoyl dehydrogenase (*DLD*, E3; EC 1.8.1.4) [[2], [3], [4]]. E3 is a common third subunit in the mitochondrial α-keto acid dehydrogenase complexes, comprising the KGDHc, the pyruvate dehydrogenase complex (PDHc), the branched-chain alpha-keto acid dehydrogenase complex (BCKDHc), and the alpha-ketoadipate dehydrogenase complex (KADHc), which catalyze − with analogous reaction mechanisms − the oxidative decarboxylation of the respective α-keto acids (for review, see Ref. [5]).

The KGDHc not only generates NADH for the electron transfer system (ETS) but also produces succinyl-CoA for mitochondrial substrate-level phosphorylation (SLP). This latter process is catalyzed by succinyl-CoA ligase (SUCLG; EC 6.2.1.4 - GTP-forming and SUCLA, EC 6.2.1.5 - ATP-forming); it becomes the primary source of ATP/GTP when oxidative phosphorylation (OXPHOS) is compromised, like in hypoxia or ETS impairment. [6,7]. α-KG also participates in various mitochondrial and cytosolic processes, like amino acid metabolism and transamination, and it serves as a substrate for α-KG-dependent dioxygenases, which play roles in DNA and histone methylation, as well as in adapting to hypoxia [[8], [9], [10], [11], [12], [13]]. Beyond its role in energy production, the KGDHc is also capable of generating reactive oxygen species (ROS) in a moonlighting enzymatic reaction, governed by the mitochondrial NADH/NAD^+^ ratio [[14], [15], [16], [17]]; remarkably, the KGDHc is also sensitive to ROS [15,[18], [19], [20]]. Consequently, *via* regulating α-KG levels, cellular processes like cell differentiation and proliferation may be controlled [9,21]. Given the above diverse functionalities, mutations in and dysfunctions of α-KG-processing enzymes are linked to various diseases, including selected neurodegenerative disorders and cancer [11,[22], [23], [24], [25], [26], [27], [28]]. Homozygous or compound heterozygous mutations in the KGDHc often lead to rare autosomal recessive genetic diseases. Acute episodes of these conditions are generally characterized by metabolic decompensations and severe lactic acidosis, which eventually lead mainly to neurological, hepatological, and cardiological symptoms [[29], [30], [31], [32]], for review see: [[5], [27], [33]]. Among these conditions, the (dihydro)lipoamide dehydrogenase (or E3) deficiency is the most severe one, since the E3 subunit (*DLD*) is shared by five different enzyme complexes. E3 deficiency is often associated with neonatal onset and premature death [27,29,30,33]. Moreover, mutations in the *DLST* gene have been associated with neurodegenerative diseases, including Alzheimer's disease (AD) and Parkinson's disease (PD) in different populations around the world [[34], [35], [36], [37], [38], [39]]. Kanamori and colleagues also identified an alternatively spliced product protein encoded by the *DLST* gene, which may play a role in the assembly of respirasome [40].

Mice with heterozygous knockout in the *DLST* (*DLST*^+/−^) or *DLD* (*DLD*^+/−^) gene have been generated to study the roles of the KGDHc in cellular metabolism both *in vitro* and *in situ* [[41], [42], [43], [44]]. Homozygous knockout of these individual genes leads to *in utero* death, indicating the essential functions of the KGDHc [43,44]. Previously, we [41] and others [7,15,16] demonstrated in isolated brain mitochondria from single heterozygous DLST^+/−^ or DLD^+/−^ mice that (*1*) the α-KG-supported O_2_ flux declined as compared to 10.13039/100010269WT animals, (*2*) the α-KG-initiated H_2_O_2_ production was reduced in the presence of a Complex I (CI) inhibitor, (*3*) the reverse electron transfer (RET)-induced H_2_O_2_ formation with succinate was diminished when compared to the controls suggesting the contribution of the KGDHc to the ischaemia-reperfusion injury [45,46], and (*4*) the expression of antioxidant enzymes declined relative to 10.13039/100010269WT pointing to the attenuation of ROS-generation by the KGDHc [7,16,41].

Previous studies on experimental animals showed that the heterozygous knockout of either *DLST* or *DLD* do not lead to phenotypic changes [7,16,44], whereas the effects of a double-heterozygous knockout (DKO, in *DLST plus DLD*) was never investigated before. Therefore, our objective here was to correlate the *in vitro* mitochondrial bioenergetic profile of the DKO mice with *in vivo* metabolic, neuronal and behavioral outcomes.

The findings in this study align well with previous research [7,16,41] that demonstrated decreased O_2_ flux in α-KG-energized mitochondria in transgenic animals. Additionally, former studies [[16], [41]] also reported a reduced RET-induced H_2_O_2_ formation rate in the transgenic mice, underpinning the ROS-producing properties of the KGDHc and suggesting its involvement in the ischaemia-reperfusion injury. Importantly, the exact subunit composition ratio of the mammalian KGDHc is still unclear. However, most research groups hypothesize a 24:24:12 E1:E2:E3 chain ratio in mammals (for review see Ref. [25] and references therein). Therefore, it is uncertain whether a combined genetic mutation of both the *DLST* and *DLD* genes would result in more than an additive effect. For the first time in the literature, we report here that middle-aged (200-250-day old) DKO animals (*1*) exhibited no significant metabolic disturbance at the whole-body level under resting conditions, (*2*) displayed decreased performance in the fatigue-endurance test, due in part to minor cardiac dysfunction, (*3*) demonstrated modest decline in cognitive abilities, (*4*) suffered from microgliosis in the cerebral cortex and neuronal cell death in the *Cornu Ammonis* subfield 1 (CA1) of the hippocampus, likely pointing to neuroinflammation, (*5*) displayed altered mitochondrial dynamics, and (*6*) produced reduced mitochondrial biogenesis. These findings demonstrate that (*1*) the animals under investigation are capable of compensating for the respective double-heterozygous knockout status with no major phenotypic alterations under resting conditions, and (*2*) that the observed *in vitro* changes correlate well with the *in vivo* alterations only under conditions of high energy demands, corroborating the presence of a dysfunctional TCA cycle.

## 2 Materials and methods

### 2.1 Animals

The heterozygous *DLD*^+/−^ (C57BL/6) mice and their wild-type (WT) littermates were purchased from the Jackson Laboratory (Bar Harbor, ME, USA). The heterozygous *DLST*^+/−^ (C57BL/6 and 129SV/EV hybrid) and WT littermate mice were obtained from Lexicon Pharmaceuticals, Inc. (Woodlands, TX, USA). Heterozygous *DLD*^+/−^/*DLST*^+/−^ double-knockout mice were generated in house *via* crossing *DLD*^+/−^ and *DLST*^+/−^ mice. 100-150- and 200-250-day old male mice were used throughout this study. Mice were housed at the minimal disease level under standard environmental conditions: 20–22 °C, 12-h light/dark cycles and 65 % humidity. Animals were fed with standard pellet chow (55 % carbohydrate, 27 % protein, 7 % lipid) optimal for rodents, especially for mice. Food and water were available *ad libitum*. Experiments were compiled in accordance with the International Guiding Principles for Biomedical Research Involving Animals and Guidelines for Animal Experiments at Semmelweis University according to the EU Directive “Directive 2010/63/EU of the European Parliament and of the Council of September 22, 2010 on the protection of animals used for scientific purposes”. All procedures and experiments were approved by the Animal Care and Use Committee of the Institute of Experimental Medicine and the Animal Health and Food Control Station, Budapest (permission numbers: PE/EA/00957-6/2024 and PE/EA/00960-4/2024). In every experiment, the experimenter/operator was blinded to the genotype of the animals.

### 2.2 Animal sacrifice and blood sample collection

Animals received i.p. heparin (5000 U/kg, Ratiopharm GmbH, Ulm, Germany) to avoid blood clotting and 3 min later were anesthetized with i.p. ketamine-xylazine (80-4 mg/kg, CP-Pharma Handelsgesellschaft GmbH, Burgdorf, Germany). To check the correct plane of anesthesia, loss of motor response (paw withdrawal) to a noxious stimulus (pinch the paw with atraumatic forceps) was performed [47]. Blood was then collected retro-orbitally (∼0.55 ml). One drop of the total blood was used for dried blood spots (DBS), the rest was centrifugated for 10 min at 1500*g* and 4 °C and sera were stored at −80 °C until use. Afterwards, the chest was opened, the right atrium was incised, and the mice were perfused transcardially with 4 °C-cold phosphate buffered saline through the left ventricle.

### 2.3 Genotyping

Polymerase Chain Reaction (PCR) quality mouse genomic DNA was isolated with a brief incubation in hot sodium hydroxide and pH adjustment with a Tris solution (HotSHOT) method as described by Truett [48]. PCR amplification was carried out by conventional polymerase chain reaction in 20 μl reaction mixture containing 1 × 10 × DreamTaq Buffer, 1 μM of each primer (wild type, mutant and common), 0.25 mM deoxynucleotide triphosphate, 0.5 U DreamTaq Green DNA Polymerase, and 2 μl template DNA (approximately 15 μg/mL). PCR reagents were obtained from Thermo Fisher Scientific (Waltham, Massachusetts, US). All oligonucleotides used in our study were procured from Sigma-Aldrich Co. and Life Technologies and are listed in Supplementary Table 1. DNA samples were subjected to thermal cycling for heterozygous *DLD*^+/−^ mice as follows: initial denaturation at 95 °C for 2 min followed by 35 cycles at 94 °C for 30 s, 61.5 °C for 60 s and 72 °C for 60 s, with a final extension of 72 °C for 7 min. For heterozygous *DLST*^+/−^ mice, the cycle conditions were as follows: initial denaturation at 95 °C for 2 min followed by 30 cycles at 94 °C for 30 s, 55.0 °C for 30 s and 72 °C for 40 s, with a final extension at 72 °C for 7 min.

### 2.4 Isolation of brain mitochondria

Mitochondria were isolated from young and middle-aged male mouse brains using a discontinuous Percoll gradient, as described earlier [41,[49], [50], [51]]. Brains were removed from the skulls and immediately transferred into ice-cold Buffer A (in mM: 225 mannitol, 75 sucrose, 5 HEPES, 1 EGTA, pH 7.4), then cut into small pieces and homogenized using a glass/Teflon Potter-Elvehjem homogenizer. The homogenate was centrifuged for 3 min at 1300*g*, the pellet was discarded, and the supernatant was centrifuged for 10 min at 20,000*g*. Afterwards, the pellet was suspended in 15 % Percoll solution and layered on a discontinuous gradient consisting of 40 and 23 % Percoll layers. This was then centrifuged for 8 min at 30,700*g*, followed by removal of the mitochondrial fraction accumulating between the 23 and 40 % Percoll layers. The mitochondrial homogenate was resuspended in Buffer A and centrifuged at 16,600*g* for 10 min. Thereafter, the pellet was again resuspended in Buffer A and recentrifuged at 6300*g* for 10 min. The supernatant was discarded, and the pellet was resuspended again now in Buffer B (in mM: 225 mannitol, 75 sucrose, 5 HEPES, pH 7.4) yielding ∼30 mg/ml mitochondrial protein content. Mitochondria were always used within 4 h after careful resuspension. All procedures were carried out in an ice-bath or at 4 °C.

### 2.5 Isolation of kidney mitochondria

Mitochondria were isolated from young or middle-aged male mouse kidneys according to Fernández-Vizarra et al. [52], with minor modifications. Kidneys were first removed, the connective tissues and the capsule were removed, then the wet mass was determined using a blotting paper. Next, the tissue was cut into small pieces with sharp scissors in ice-cold isolation Buffer C (in mM: 220 sucrose; 70 mannitol, 10 EGTA, 2 HEPES, pH 7.4), and then washed in the same buffer to remove the remaining blood. Afterwards, the tissue was homogenized with a Dounce homogenizer and then centrifuged at 900*g* for 10 min. The supernatant was then centrifuged at 15,000*g* for 5 min. Next, the supernatant was discarded, the pellet was resuspended in ice-cold isolation Buffer C (10 ml/g wet tissue) and transferred into two Eppendorf tubes for further centrifugation at 15,000*g* for 5 min twice. Finally, the pellet was resuspended in 75 μl isolation Buffer C yielding ∼40 mg/ml mitochondrial protein content. All procedures were carried out in an ice-bath or at 4 °C. Mitochondria were always used within 4 h after careful resuspension.

### 2.6 Determination of protein concentration

The mitochondrial protein concentration was determined by using a modified Biuret assay [53,54].

### 2.7 Respiratory medium

Mitochondrial oxygen consumption, H_2_O_2_ production, and ATP generation were all monitored in a respiratory buffer containing (in mM) 125 KCl, 20 HEPES, 2 KH_2_PO_4_, 0.1 EGTA, 1 MgCl_2_ and 0.025 % fatty-acid free bovine serum albumin (BSA), pH 7.4.

### 2.8 Mitochondrial oxygen consumption

Mitochondrial oxygen consumption was recorded using high-resolution respirometry (Oroboros Instruments, Innsbruck, Austria) at 37 °C in 2-mL chambers under continuous stirring (750 rpm) [55]. Oxygen concentration was monitored by the polarographic oxygen sensor (POS) and recorded *via* the program DatLab7.4 (Oroboros Instruments). POS was calibrated daily at air saturation, routinely in oxygen-depleted media. Instrumental O_2_ background tests were performed periodically. O_2_ fluxes were calculated real-time, as the negative time derivative of the O_2_ concentration, and corrected for (*1*) the instrumental background O_2_ flux, (*2*) sample dilution by titrations, and (*3*) residual oxygen consumption measured in the presence of isolated mitochondria but no ADP or any respiratory fuel substrates, all in DatLab7.4. Mitochondria (0.05 mg/ml) were energized with either α-ketoglutarate (α-KG; 5 mM) or succinate (10 mM) to measure the LEAK respiration. Then, saturating concentration of ADP (2.5 mM) was added to measure the OXPHOS capacity.

### 2.9 Mitochondrial ATP production

The mitochondrial ATP generation rate was measured by using a coupled-enzyme assay that applies hexokinase (HK) and glucose-6-phosphate dehydrogenase (G6PDH) [56,57]. The respiratory medium was supplemented with 2.75 mM glucose, 1.65 mM NADP^+^, 1.1 U G6PDH, 4.4 U HK, and 150 μM P^1^,P^5^-di(adenosine-5′)-pentaphosphate (AP5), an inhibitor of adenylate kinase, in 2 ml total volume [56]. In the presence of respiratory substrates and ADP, mitochondria produced ATP that left the mitochondria *via* the adenine nucleotide translocase (ANT). In the extramitochondrial medium, glucose got oxidized to glucose-6-phosphate by HK using the previously produced ATP. Then, glucose-6-phosphate got converted to 6-phosphogluconate by G6PDH with the concomitant reduction of NADP^+^ to NADPH. The generation of NADPH was recorded at 340 nm and 37 °C using a JASCO V-650 spectrophotometer (ABL&E-JASCO, Tokyo, Japan). The NADPH absorbance (ε = 6220 l ∙ mol^−1^ ∙cm^−1^]) was proportional to the amount of ATP released from the mitochondria. The assay was calibrated using ATP standards (100 μM). Mitochondria (0.05 mg/ml) were energized with either α-KG (5 mM) or succinate (10 mM), which was followed by ADP (2.5 mM) addition to induce ATP production. Then, oligomycin (25 nM) was added to inhibit OXPHOS *via* the inhibition of the ATP-synthase. At the end, FCCP (25 nM), an uncoupler, was administered to measure SLP.

### 2.10 Detection of mitochondrial reactive oxygen species

Hydrogen peroxide formation was monitored using the Amplex UltraRed assay [49,58,59]; horseradish peroxidase (HRP; 2.5 U/ml), Amplex UltraRed (2 μM), and mitochondria (0.1 mg/ml) were added to the respiratory medium. Fluorescence was recorded at 550 nm excitation and 585 nm emission wavelengths in a PTI Deltascan fluorescence spectrophotometer (Photon Technology International, Lawrenceville, NJ, USA) at 37 °C. The fluorescence signal was calibrated with H_2_O_2_ (100 pmol) at the end of each experiment. The H_2_O_2_ fluxes were normalized to the background flux measured in the presence of mitochondria. Measurements were carried out at air saturation (∼190 μM O_2_) in an open cuvette. H_2_O_2_ production by kidney mitochondria was monitored in the presence of 100 μM phenylmethyl sulfonyl fluoride (PMSF), which is an inhibitor of carboxylesterases. According to Ref. [60,], carboxylesterases may convert Amplex UltraRed to the end product, fluorescence dye resorufin requiring no HRP, O_2_, or H_2_O_2_ leading to fluorescent artifacts. As a control, H_2_O_2_ generation was also recorded using high-resolution respirometry (Oroboros Instruments, Innsbruck, Austria) in parallel with measuring the mitochondrial O_2_ consumption in the same measurement chamber close to air saturation (∼190-150 μM O_2_) and at tissue normoxia (∼80-30 μM O_2_) in the KCl-based respiratory medium and in MiR05 (Oroboros Instruments) having high antioxidant capacity (data not shown) [58,61,62]. In both respiration media, we observed identical tendencies for H_2_O_2_ production in the animal groups investigated (data not shown).

### 2.11 Enzyme activity assays

Experiments for enzyme activity were carried out in 50 mM KH_2_PO_4_, pH 7.4 applying 0.1 mg/ml mitochondria; traces were recorded with a JASCO V-650 spectrophotometer (ABL&E-JASCO, Tokyo, Japan). The final results were calculated with the theoretical molar extinction coefficient from the literature for NAD(P)H, ε = 6220 l ∙mol^−1^ ∙cm^−1^.

Aconitase activity was measured *via* spectrophotometry as previously described [19,63]. Briefly, the medium was supplemented with 2 mM EGTA, 3 mM MgCl_2_, 0.6 mM MnCl_2_, 0.1 % v/v Triton X-100, and 0.281 U NADP^+^-dependent isocitrate dehydrogenase (ICD), which was followed by the addition of the mitochondria. The reaction was launched by the addition of 0.5 mM NADP^+^ and 10 mM citrate, while NADPH formation was monitored at 340 nm.

The overall KGDHc activity was measured *via* again spectrophotometry as before with minor modifications [19,64]. Briefly, the medium contained 2 mM EGTA, 3 mM MgCl_2_, 0.2 mM TPP, 2.5 mM ADP, 2 mM NAD^+^, 1 μM rotenone, 0.1 % v/v Triton X-100, and mitochondria and the reaction was commenced *via* administering 5 mM α-KG and 0.12 mM CoA-SH. NADH formation was monitored at 340 nm.

### 2.12 mRNA expression analysis

Total RNA was extracted from liver tissues using the Direct-zol™ RNA Miniprep Plus Kit according to the manufacturer's instructions (Zymo Research, Irvine, CA, US). Total RNA (1 μg) from each sample was used to prepare cDNA with an oligo-dT primer by single-strand reverse transcription (RevertAid First Strand cDNA Synthesis Kit, Thermo Fisher Scientific, Waltham, Massachusetts, US). Gene expression was analyzed *via* quantitative real-time PCR using the Power SYBR Green PCR Master Mix (Life Technologies, Carlsbad, CA, US) and an Applied Bioscience QuantStudio 12K Flex Real-Time PCR system (ThermoFisher Scientific). We used 96-well plates with a reaction volume of 10 μl, according to the manufacturer's instructions. Relative mRNA quantification was performed by using the 2^−dCt^ method and normalizing to ribosomal protein 29 (Rps29). All reactions were carried out in duplicates. The gene-specific primers are listed in Table 1 [65].

**Table 1:** Gene-specific primers used for mRNA expression.

Gene	Forward (5’ ->3′)	Reverse (5’->3′)
Glutamate dehydrogenase 1	GCTAAAGCAGGCGTTAAGA	TCAATGCCAGGACCAATAAA
α-ketoglutarate dehydrogenase (E1k)	CAACTCAGATGACCCTGAAG	GTCGATAACACACCAGATCAA
Dihydrolipoamide dehydrogenase (E3)	GCTTGAACGTTGGTTGTATTC	TCAAGCGAACTTCTGGTATTT
Dihydrolipoamide succinyltransferase (E2k)	CTTCAGGGTTCGCTTCTTC	GCATCTCCAACAGCTTTCT
Pyruvate carboxylase	CAGAGGAGTTTGAGGTTGAG	GAAGCTGCCCATTGAGTT
ATP citrate:lyase	GGGAGGAAGCTGATGAATATG	GTCAAGGTAGTGCCCAATG
Branched-chain alpha-keto acid dehydrogenase (E1b)	CCAGGGATCAAGGTGGTAATA	TGCTGCCCGGTAAAGTAT
Succinate dehydrogenase complex, subunit A	CAAGACTGGCAAGGTTACTT	TCATCAGTAGGAGCGGATAG
Ribosomal protein S29	CGGTCTGATCCGCAAATAC	GGTCGCTTAGTCCAACTTAAT

### 2.13 Western blot analysis

Pellets of isolated mitochondria from WT and DKO mice were resuspended in radioimmunoprecipitation assay (RIPA) buffer (10 mM Tris/HCl, pH 7.4, 1 % NP-40, 0.1 % sodium deoxycholate, 0.1 % SDS, 150 mM NaCl) in the presence of protease inhibitor mix (P8340-5 ml). After 20 s sonication (Sonopuls HD 2070 probe sonicator, Bandelin electronic GmBH & Co. KG, Berlin, Germany) experiments were carried out on ice, cycle interval was 15 s, power amplitude was 10 %, the mitochondrial debris was removed by centrifugation at 14,000*g* and 4 °C for 10 min. Protein concentrations were determined from the supernatants with the bicinchoninic acid (BCA) assay using bovine serum albumin (BSA) as standard. 10 μg protein samples were denatured for 5 min at 95 °C, then loaded on a non-reducing 10 % polyacrylamide gel. After size separation by electrophoresis at 140 V, proteins were transferred to a nitrocellulose membrane for 15 min (Trans-Blot Turbo Blotting System, Bio-Rad, Hercules, CA, USA). Transfer efficiencies were assessed *via* Ponceau staining. Membranes were blocked for 1 h at room temperature (RT) in Tris-buffered saline with Tween-20 (TBST; 20 mM Tris, 150 mM NaCl, pH 7.4, 0.05 % Tween-20) containing 5 % non-fat milk and 0.5 % BSA. Primary antibodies against E1k (ab137773), E2k (ab177934) and E3 (ab133551) were purchased from Abcam (Cambridge, UK). The primary antibodies against Complex IV (CIV; 4850T) were from Cell Signaling (Danvers, MA, USA). The antibodies were diluted into TBST and incubated with the nitrocellulose membrane overnight at 4 °C. After thorough washing in TBST (3 × 10 min), the membranes were incubated with horseradish peroxidase-conjugated anti-rabbit IgG secondary antibodies (Enzo Life Sciences, Farmingdale, NY, USA) in 1:5000 dilution in TBST for 1 h at RT. After another round of TBST washing for 3 × 10 min, the membranes were incubated with the ECL reagent (Bio-Rad, Hercules, CA, USA) for 2 min and the signal was detected using a gel documentation system (Syngene, Cambridge, UK). Immunoblotting for CIV was applied to normalize protein expression levels. Densitometry was performed using the software ImageJ. The primary antibodies were used in the following dilution ratios: E1k – 1:1,000, E2k – 1:10,000, E3 – 1:10,000, CIV – 1:1000.

### 2.14 Determination of body composition

Total body composition was determined with an EchoMRI device (700 Whole Body Composition Analyzer; E26-233RM, Echo Medical Systems, Houston, TX, USA), before the metabolic measurements.

### 2.15 Determination of metabolic parameters

Mice were singly housed for one week before the experiments. Two days before the metabolic measurements, mice were transferred to test cages of TSE Phenomaster setup (TSE Systems, Berlin, Germany) to acclimate to the measurement environment and to learn the usage of drinking bottles and feeders. The metabolic variables (food and water intake, X-Y-Z locomotor activity, O_2_ consumption and CO_2_ production) were automatically recorded in every 15 min for 24 h [66]. The respiratory exchange ratio (RER) was calculated with the following formula: V_CO2_/V_O2_. Afterwards, the same metabolic parameters were recorded for 24 h at thermoneutral temperatures (29–30 °C).

### 2.16 Open field test

The open field test is an experimental test to study vertical and horizontal locomotor activity levels, anxiety, and willingness to explore [67]. The open field test box consisted of a circular arena 80 cm in diameter, which was subdivided into 20 subsectors by concentric and radial lines and surrounded by a 35 cm high aluminium wall. Each animal was placed in the center of the open field and their psychomotor activities were assessed by visual observation for 5 min. The horizontal movement activity was evaluated *via* line crossings by walking, whereas the vertical movement activity was judged by rearings. The anxiety level was defined according to the time spans spent in the inner and outer zones. The arena was cleaned with 70 % v/v ethanol and a dry paper towel between animal tests.

### 2.17 Novel object recognition test

Novel object recognition was tested in an open field arena one day after the open field test, as previously described [68,69]. During the first trial (sample trial), two identical objects were placed in the arena at equal distances from the wall in an asymmetric geometry relative to the center. The mice were allowed to explore these objects for 5 min and then they were removed from the arena and placed in a cage. After 120 min, during the second trial, one of the previous objects was replaced with a novel object (test trial). The animal explored the two objects for 5 min. The total number of visits (frequency) and the time spans spent *per* visit at each object were recorded in seconds. A recognition index (RI) in percentage was calculated to evaluate the behavioral performance in recognizing the new object as opposed to the familiar one. The following equation was used for this purpose: RI = [duration of visits to novel object/(duration of visits to novel *plus* familiar objects)] × 100.

### 2.18 Morris water maze test

The Morris water maze test is an experimental test to examine the spatial learning capacity of animals [70]. The test was carried out in a round-shaped black water tank (diameter: 153 cm, height: 63 cm) filled to a depth of 53 cm with water of 24 ± 1 °C. A black hidden platform (diameter: 10.8 cm) was submerged at a fixed position 1.5 cm below the surface of the water. Animals were tested on five consecutive days in five sessions (one session *per* day) with different starting positions around the perimeter of the tank. Each session was conducted with four trials with various orders of starting positions. Each trial lasted until the mouse found and located the platform. If the platform was not found within 90 s, the experimenter placed the animal on it. At the end of each trial, the mouse spent 30 s on the platform. The time to find the platform was measured in each trial and registered as latency in seconds. During each session, the latency time of the first trial served as reference memory (RM) recording, whereas the mean latency time of the daily four trials as working memory (WM) counting.

### 2.19 Treadmill fatigue test

Before performing the fatigue test, animals were trained for two days to use the treadmill [71]. On the first day, the mice explored the treadmill for 1–3 min, then the treadmill was turned on and the speed slowly increased up to 8 m/min. At 5 min, the speed was increased to 9 m/min and at 7 min to 10 m/min, whereas the treadmill was stopped at 10 min. On the second day, after exploration, the speed levelled up to 11 m/min at 5 min and to 12 m/min at 10 min. Then, the treadmill was stopped at 15 min. On the third day, the speed was set to 12 m/min and then increased according to Table 2.

**Table 2:** Treadmill speed as a function of time.

Time (min)	Speed (m/min)
0.5	14
1	16
6	18
30	20
45	22
60	24
75	26

In cases when the mouse stayed in the fatigue zone (the rear of the treadmill, ranging from approximately one body length from the grid) for five consecutive seconds, it got removed from the treadmill instantaneously.

### 2.20 Blood tests

Amino acid and acylcarnitine profiles in mouse DBS were determined using a non-kit derivatization method [72] by electrospray tandem mass spectrometry (ESI–MS/MS) on a Sciex Triple Quad 3500 system (Sciex, Framingham, USA). Amino acid analyses from mouse sera were performed using the MassChrom® Amino Acid-LC-MS/MS kit from Chromsystems (Gräfelfing, Germany) on a QTRAP 4500 LC-MS/MS System (Sciex, Framingham, USA), according to the vendor's specifications.

### 2.21 Echocardiography

Echocardiography was performed using a Vevo 3100 imaging system (Fujifilm VisualSonics, Toronto, Canada) with an MX400 transducer. Mice were anesthetized with isoflurane (5 % for induction, 2 % for maintenance) and placed on a heating pad to maintain physiological temperature. Animal core temperature was monitored throughout the whole procedure with a rectal thermometer. Heart rate was monitored using electrocardiography. Measurements were performed as described earlier [73] in accordance with the international standards [74]. Two-dimensional long-axis measurements were used to measure end-diastolic and end-systolic left ventricular (LV) volumes (LVEDV and LVESV, respectively). Short-axis measurements were applied to measure end-diastolic and end-systolic left ventricular diameters (LVIDd and LVIDs, respectively) and LV anterior and posterior wall thicknesses (LVAWs, LVAWd, LVPWs and LVPWd, respectively). Early mitral valve peak blood-flow velocity (E), early mitral annulus peak movement velocity (e’) and isovolumetric relaxation time (IVRT) were measured with doppler measurements in apical four chamber imaging plane.

Stroke volume (SV) was calculated as LVEDV - LVESV. Ejection fraction (EF) was determined as SV/LVEDV × 100. Cardiac output was calculated as SV × HR. Fractional shortening (FS) was calculated as (LVIDd–LVIDs)/LVIDd. Left ventricular mass was estimated using the following formula: 1.053 × [(LVIDd + LVAWd + LVPWd)^3^–LVIDd^3^] × 0.8. The left ventricular remodeling index was calculated as left ventricular mass/LVIDd. Echocardiographic recordings were evaluated *via* the software VevoLAB (Fujifilm VisualSonics, Toronto, Canada). The investigator and the analyzer were blinded to the experimental groups.

### 2.22 Histology

Mouse brains were fixed in 4 % v/v phosphate-buffered formaldehyde for 24 h at RT (25 °C), then were desiccated in graded alcohol series and embedded in paraffin blocks. 5-μm thick sections were made by a microtome (HistoCore MULTICUT, Leica Biosystems, Nussloch, Germany) while preparing for immunohistochemistry. After deparaffinization in xylene for 2 × 10 min, the histology slides were rehydrated and then subjected to heat-induced epitope retrieval at pH 9 and 95 °C for 30 min. Aspecific protein binding was reduced by 5 % skimmed milk in Tris-buffered saline solution. Tissues were incubated with specific antibodies for 1 h at RT. Incubations with secondary HRP-conjugated antibodies were performed for 1 h at RT. Each step was divided with washing in Tris-buffered saline solution for 3 × 5 min at RT. 3,3′-diaminobenzidine (DAB) was utilized as chromogen to reveal antibody labeling. Staining intensity was quantified with the H-score method, as follows: H-score=(weak intensity % × 1)+(medium intensity % x2)+(strong intensity % × 3). That means what percentage of the area on the slice stained with weak, medium or strong intensity. The maximum value is 300, while the minimum value is 100. Utilized primary antibodies: Anti-CD-68 polyclonal antibody (dilution: 1:500; 25747-1-AP, PTG labs, Manchester, UK), anti-PGC1A (dilution: 1:100; (66369-1-Ig, PTG labs, Manchester, UK), anti-Nrf2 antibody (dilution: 1:100; 16396-1-AP, PTG labs, Manchester, UK), anti-DRP1 antibody (dilution: 1:100; 12957-1-AP, PTG labs, Manchester, UK), anti-MFN2 antibody (dilution: 1:100; 12186-1-AP, PTG labs, Manchester, UK), anti-mTFA antibody (dilution: 1:100; ab307302, AbCam, Cambridge, UK). Secondary antibodies: Rabbit IgG VisUCyte HRP Polymer Antibody (VC003–050, R&D Systems, Inc, MN), Mouse IgG VisUCyte HRP Polymer Antibody (VC001–050, R&D Systems, Inc, MN), DAB (Vector Labs, Redcar, UK). Skeletal muscle samples from the musculus quadriceps femoris were prepared with the routine formalin-fixed then paraffin-embedded process (see above). The sections were 5 μm thick and stained with haematoxylin and eosin for histological estimation; massive necrosis, inflammation or fatty degeneration were inspected. Sirius red stain (Thermo Fischer Scientific) was also applied to reveal any sign of fibrosis. The sections were stained with picrosirius red stain (PanReac AppliChem, Darmstadt, Germany) for 1 h at RT, then the samples were washed twice with acidified water (0.5 % v/v acetic acid) and in the end sections were dehydrated with pure (96% v/v) ethanol and cleared with xylene.

### 2.23 PET-CT

A volume of 0.18 ± 0.09 ml FDG (activity of 9.39 ± 1.25 MBq; means ± SD) was administered intravenously into the lateral tail vein. Mice were anesthetized with isoflurane (1.5 %) for the whole duration of imaging with CT and PET (nanoScan SPECT/CT and nanoScan PET/MRI -3T; Mediso, Hungary). Helical CT scan settings: 50 kVp and 980 μA, 300 ms experimental time, 1:4 binning, pitch: 1, zoom factor: 1.2, 687 projections (360 projections/rotation). Static scans of 60-min duration were collected 10 min post-injection in an energy window of 400–600 keV. All PET images were reconstructed using the respective device's three-dimensional Maximum A Posteriori (MAP, P4 system) or three-dimensional Monte-Carlo-modeled Ordered Subsets Expectation Maximization (Tera-Tomo 3D-OSEM, Mediso, Hungary) algorithm, with corrections for dead time, scattering, and attenuation, where available. Reconstructed images of 0.3 mm voxel size were then visualized using the softwares VivoQuant (inviCRO, USA) and Fusion (Mediso, Hungary), respectively. To assess energy consumption, we utilized the standardized uptake values (SUVs).

### 2.24 Chemicals

All chemicals, unless specified otherwise, were obtained from the Merck Group (Darmstadt, Germany), except for Amplex UltraRed and the RevertAid First Strand cDNA Synthesis Kit (both from ThermoFisher Scientific, Waltham, MA, US), Direct-zol™ RNA Miniprep Plus Kit (Zymo Research, Irvine, CA, US), and Power SYBR Green PCR Master Mix (Life Technologies, Carlsbad, CA, US).

### 2.25 Statistics

Statistical analyses were performed with the software GraphPad Prism 8.0. Data are expressed as means ± S.E.M. Statistical differences were analyzed by two-way ANOVA followed by Sidak's multiple comparisons test. The unpaired *t*-test was used when two groups were evaluated.

## 3 Results and discussion

1 Overall KGDHc activity, mRNA/expression levels of E2k and E3 in DKO mouse mitochondria

**Fig. 1:**
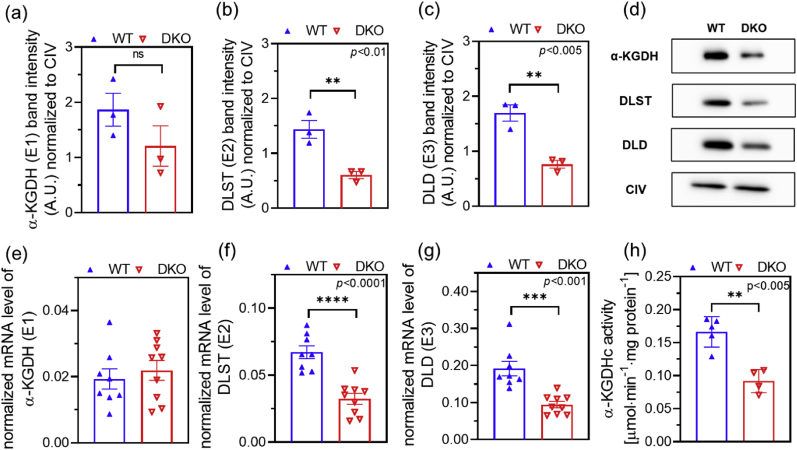
**Western blot analyses for the E1k (a), E2k (b) and E3 (c) proteins in the liver of WT and DKO mice.** Representative images are shown in (d). Band intensities were normalized to ETS CIV expression. Relative mRNA levels for the E1k (e), E2k (f) and E3 (g) proteins in mouse liver. mRNA levels were normalized to ribosomal protein S29 (Rps29). The overall KGDHc enzyme activity (h) was measured in isolated brain mitochondria. Data are shown as means ± S.E.M. and were analyzed by the unpaired *t*-test; n = 3–9/groups.

As was expected, both the protein and mRNA levels of E2k and E3 reduced in the liver of DKO mice as compared to controls (Fig. 1): these reductions were 58 and 45 % for E2k, whereas 55 and 46 % for E3, respectively. We also observed a moderate reduction in the E1k protein level (35 %), which was also found earlier by us in the single heterozygous *DLST* and single heterozygous *DLD* KO mice [41]; remarkably, the E1k mRNA level displayed no variation in the three types of probands. As was again expected, the overall KGDHc activity significantly decreased, by 45 %, in isolated brain mitochondria of the DKO animals.

2 O_2_ consumption and ATP production in α-KG- or succinate-supported DKO mouse mitochondria

**Fig. 2:**
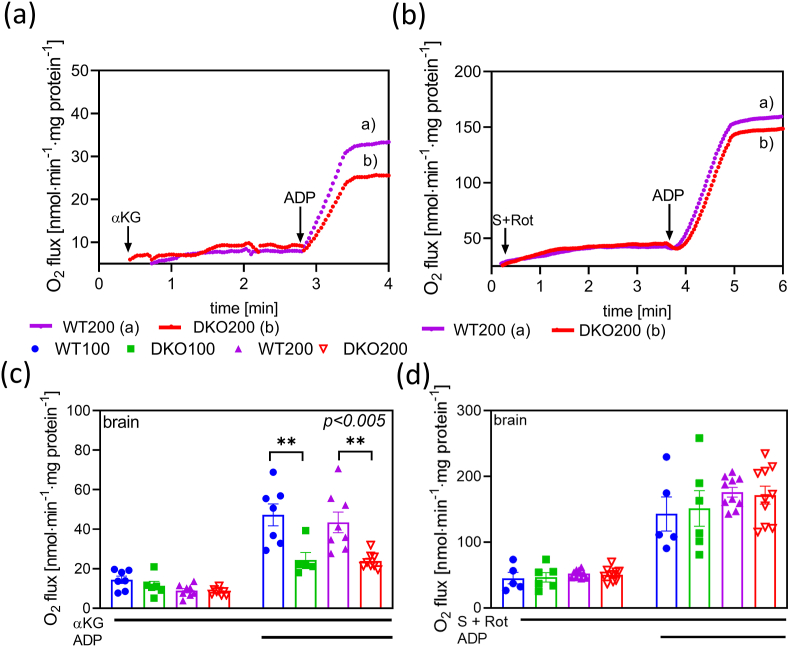
**O_2_ consumption in isolated brain mitochondria energized with either α-KG or succinate in four different animal groups.** Representative traces of independent experiments are shown in panel (a) using α-KG and (b) with succinate in brain mitochondria. Mitochondria were incubated in respiratory medium as described under Materials and Methods. α-KG or Rot and succinate, and ADP were added as indicated. The O_2_ consumption is represented as means ± S.E.M. (c–d). Data were analyzed by two-way ANOVA followed by Sidak's multiple comparison test; n = 5–10/group. Blue circle - WT100: 100-150-day old WT mice; green square – DKO100: 100-150-day old DKO mice; purple triangles – WT200: 200-250-day old WT mice; open red triangles – DKO200: 200-250-day old DKO mice. Lines under the panels indicate additions of chemicals to the mitochondrial suspension.

**Fig. 3:**
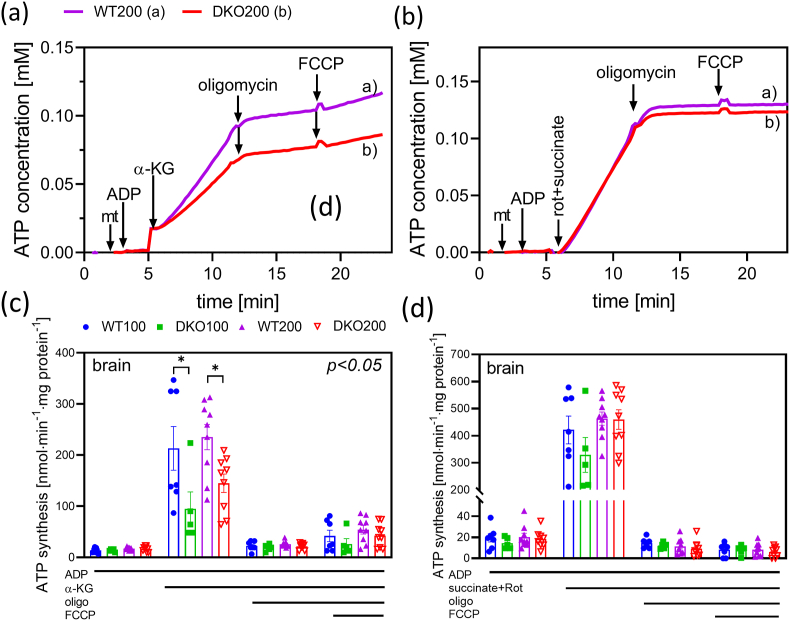
**ATP synthesis in isolated brain mitochondria energized with either α-KG or succinate in the four different animal groups.** Representative traces of independent experiments are shown in panels **a** (α-KG) and **b** (succinate). Mitochondria were administered into the respiratory medium after HK, glucose, NADP^+^, G6PDH and AP5 were given. Thereafter, ADP, respiratory substrates (α-KG, or Rot and succinate), oligomycin and FCCP were added, as indicated. ATP synthesis is expressed as means ± S.E.M. (c–d). Data were analyzed by two-way ANOVA followed by Sidak's multiple comparison test; n = 5–9/group. Lines under the panels indicate additions of chemicals to the mitochondrial suspension.

Any impairment in the ETS or TCA cycle is typically reflected in changes to mitochondrial O_2_ consumption and ATP production, which are sensitive indicators of the mitochondrial bioenergetic status. In isolated mitochondria, specific respiratory pathways get activated upon exogenously administered respiratory fuel substrates, in either the presence or absence of ADP. In this study, mitochondrial O_2_ consumption and ATP synthesis were measured in mitochondria respiring on either the NADH-linked substrate α-KG or succinate (Fig. 2, Fig. 3). Initially, α-KG was added in the absence of ADP to monitor LEAK respiration (Fig. 2a and c), which showed no statistically significant variations amongst the animal groups. This was followed by giving a saturating concentration of ADP to initiate α-KG-linked OXPHOS capacity, which was reduced by 43 % in the DKO200 group and by 48 % in the DKO100 group as compared to age-matched controls. In another set of experiments, the FADH_2_- (or Complex II) linked substrate, succinate, was added to the mitochondria (Fig. 2b and d). Typically, succinate-linked respiration is measured in the presence of the CI inhibitor, rotenone, to prevent oxaloacetate accumulation due to the activity of the NADH-linked malate dehydrogenase [75,76]. It is well-known that oxalacetate is an inhibitor of succinate dehydrogenase resulting in an inhibition of the succinate-linked O_2_ flux [[75], [76], [77]]. However, in the presence of rotenone, NADH accumulates and slows the formation of oxalacetate. As expected, succinate-induced O_2_ flux, with or without ADP, did not significantly differ among the WT and DKO groups (Fig. 2b and d). This is because succinate enters the TCA cycle after the reaction catalyzed by KGDHc, so succinate-initiated O_2_ consumption is not affected by the heterozygous knockout of the KGDHc. Notably, similar trends were observed in the kidney mitochondria isolated from the DKO mice (Fig. S1).

ATP synthesis in the isolated mitochondria was indirectly monitored *via* a coupled-enzyme assay, as was described under Materials and Methods. Initially, ADP and a respiratory substrate, α-KG or succinate, were given to induce OXPHOS (Fig. 3). As was expected, the α-KG-linked ATP synthesis (Fig. 3a–c) declined in the DKO mice, relative to the controls (WT100: 213 ± 40; DKO100: 95 ± 30; WT200: 235 ± 23; DKO200: 150 ± 17 nmol·min^−1^·mg^−1^). Oligomycin, an ATP-synthase inhibitor, lowered the ATP production in all the animals, confirming the dominant role of the F_o_F_1_-ATPase in mitochondrial ATP generation. An uncoupler (FCCP) augmented the rate of ATP formation by 216 ± 34 % in the WT200 group and by 188 ± 24 % in the DKO200 group, which is attributed to SLP governed by succinyl-CoA ligase [56]. This demonstrated that the TCA cycle remained somewhat functional and was still capable of producing succinyl-CoA, although in rather limited amounts, for SLP in the DKO group. The compromised overall KGDHc activity led to a lower provision of succinyl-CoA for matrix SLP in the DKO mice, relative to control, resulting in further decline in the mitochondrial ATP concentration. This gave rise to a decline in mitochondrial membrane potential (ΔΨ_m_), which may have led to the reversal of the ANT and potential utilization of extramitochondrial ATP to maintain the ΔΨ_m_ [6,7]. These fluctuations in the mitochondrial ATP level may indeed render the DKO animals more vulnerable to mitochondrial damage and various toxins [44,78].

In another set of experiments, succinate was applied as the respiratory substrate in the presence of rotenone (Fig. 3b–d). As was expected, there have been no statistically significant differences observed in the succinate-linked OXPHOS in the DKO mice, relative to the controls (WT100: 421 ± 51; DKO100: 329 ± 65; WT200: 463 ± 25; DKO200: 460 ± 36 nmol ·min^−1^·mg^−1^). Moreover, the ATP formation that was once inhibited by oligomycin could not be stimulated by FCCP, likely since the mSLP (m for mitochondrial) cannot be supported *via* succinate [7,56].

In summary, in accord with the respective findings by Kiss et al. [7], the ADP-initiated O_2_ flux and ATP production significantly declined in the DKO mitochondria when respiring on α-KG; succinate exerted no such effects. When comparing to single KO animals [7,41], the reduction in the O_2_ flux proved to be additive in the DKO mice. However, the ATP production already decreased by 50 % in the single KO mutants, similarly to the DKO mitochondria [7,79].This phenomenon may be attributed to a loosely-coupled ETS even in single KO mitochondria under *in situ* conditions.

3 α-KG- and RET-initiated H_2_O_2_ production in DKO mouse mitochondria

ROS are by-products in the aerobic respiration machinery; imbalance of their production and elimination may lead to oxidative stress [[80], [81], [82]]. ROS generation by the KGHDc is implicated in select neurodegenerative diseases; for review, see: [25]. ROS-forming sites may be traced down in isolated mitochondria *via* applying specific respiratory substrates, ETS inhibitors, and/or uncouplers. In this study, we were principally interested in the α-KG-initiated H_2_O_2_ formation upon CI inhibition and succinate-induced RET, since RET is an important source of ROS in the course of the ischaemia-reperfusion injury [45,46]. During RET, electrons flow backwards from CII towards CI, leading to high rate of ROS formation [49,83].

**Fig. 4:**
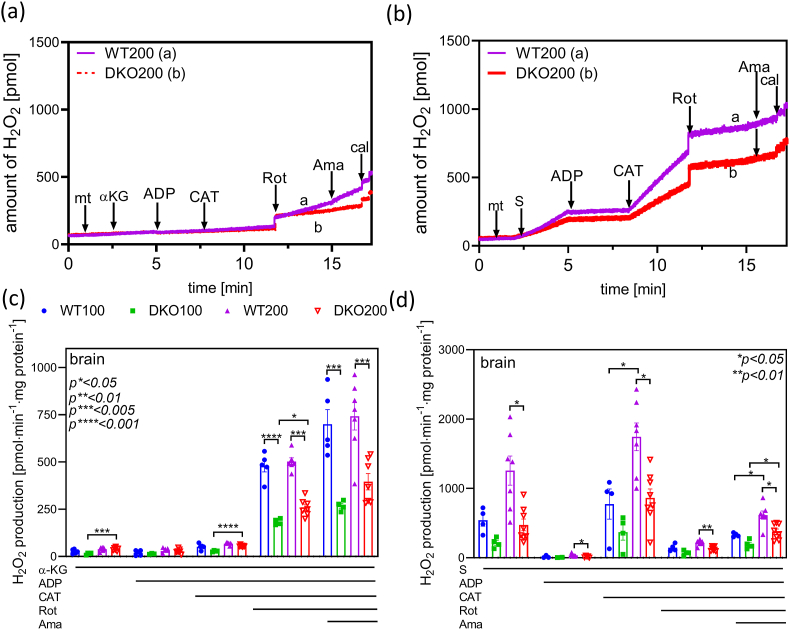
**H_2_O_2_ production in brain mitochondria energized with either α-KG or succinate in four different animal groups.** Representative traces of independent experiments are shown in panels (a**)** and (b). Mitochondria were administered into the respiratory medium after Amplex UltraRed and horseradish peroxidase (HRP). Afterwards, α-KG or succinate, ADP, CAT, Rot, and Ama were given, as indicated. H_2_O_2_ production is expressed as means ± S.E.M. (c–d). Data were analyzed by two-way ANOVA followed by Sidak's multiple comparisons test; n = 4–9/group. Lines under the panels indicate additions of the respective chemicals into the mitochondrial suspension.

In isolated brain mitochondria respiring on α-KG, a low rate of H_2_O_2_ formation could be detected, which was attributed to the KGDHc and ETS (Fig. 4a and c, and S2a). Under these conditions, no statistically significant difference could be observed between the corresponding control and DKO groups (39 ± 6 and 39 ± 4 pmol·min^−1^·mg^−1^ in the DKO200 and WT200 groups, respectively). ADP just barely affected the α-KG-induced H_2_O_2_ production in all the animal groups investigated. CAT, an ANT inhibitor, stimulated the formation of H_2_O_2_ owing to the hyperpolarization of ΔΨ_m_. Rotenone, a CI inhibitor, significantly augmented the H_2_O_2_ formation, which could in part be ascribed to CI and KGDHc due to an elevated NADH/NAD^+^ ratio (from 56 ± 8 to 206 ± 38 pmol·min^−1^·mg^−1^ in the DKO200, from 64 ± 5 to 461 ± 17 pmol ·min^−1^·mg^−1^ in the WT200 groups). The rotenone-induced H_2_O_2_ formation in the corresponding age-matched controls reduced by 62 and 44 % in the DKO100 and DKO200 animals, respectively; these findings are in accord with previous studies which were carried out with single heterozygous *DLST*^+/−^ or *DLD*^+/−^ brains [16,41]. Antimycin A (Ama) moderately enhanced the H_2_O_2_ production owing to the inhibition of the Qi site in CIII [84,85].

As for the *DLST*^+/−^ and *DLD*^+/−^ mice, an intense H_2_O_2_ generation was observed in isolated mitochondria with succinate and no ADP, which was attributed to RET (Fig. 4b and d, and S2b). The RET-induced H_2_O_2_ generation with succinate in age-matched controls decreased by 61 and 63 % in the DKO100 and DKO200 animals, respectively. These findings suggest that the KGDHc is capable of producing ROS in its reverse catalytic direction, with no α-KG, at high NADH/NAD ^+^ ratios. The RET is highly susceptible to alterations in ΔΨ_m_ [86] and since ADP induced depolarization, a decline in the RET-initiated H_2_O_2_ formation could be observed. CAT, on the contrary, hyperpolarized the mitochondrial inner membrane, which favored RET, and hence the H_2_O_2_ formation got stimulated. In the DKO100 and DKO200 animals, the CAT-induced H_2_O_2_ formation reduced by 52 and 50 %, respectively, relative to controls. Succinate *plus* CAT enhanced the RET-induced H_2_O_2_ production in the middle-aged animals of both genotypes as compared to the corresponding younger groups, pointing to an age-specific increase in the RET [87]. Rotenone inhibited the RET-mediated H_2_O_2_ generation since it binds to the CI in close spatial vicinity to the coenzyme Q-binding site, thus hindering the electron transfer through the CI [88]. Ama moderately further augmented the H_2_O_2_ generation [84,89].

As seen above, ROS formation declined in DKO animals, which could be considered as a beneficial effect. However, it may also become disadvantageous if it interferes with physiological ROS-mediated pathways. H_2_O_2_ generation decelerated in the DKO animals with α-KG or succinate, which accounts for the ROS-producing capacity of the KGDHc as well as the contribution of this enzyme complex to RET. This could also propose a protective role for the E2k/E3 inhibition in the ischaemia-reperfusion injury. In the course of an ischemic injury, succinate accumulates. As a consequence, in the early phase of reperfusion the RET, potentially besides the CI and PDHc [90], triggers ROS formation that contributes to oxidative damage [45]. Lowering the KGDHc-mediated ROS load might be a potential therapeutic avenue in combating the oxidative reperfusion injury. Further studies will be required to fully understand the pathophysiological significance of the reduced ROS generation in this animal model.

Quite importantly, no difference in CI activity could be detected between corresponding DKO and WT groups (data not shown), ruling out the possibility that the decline in H_2_O_2_ production was due to reduced CI activity. Similarly, CI and succinate dehydrogenase activities were also maintained in the brain mitochondria of single *DLD*^+/−^ mice [16]. Interestingly, the H_2_O_2_ flux was greater in the brain mitochondria of elder animals in the presence of α-KG and CAT, and during RET pointing to an age-dependent enhancement in ROS production (for review, see Ref. [91]).

4 Aconitase activity in DKO mouse mitochondria after H_2_O_2_ exposure

**Fig. 5:**
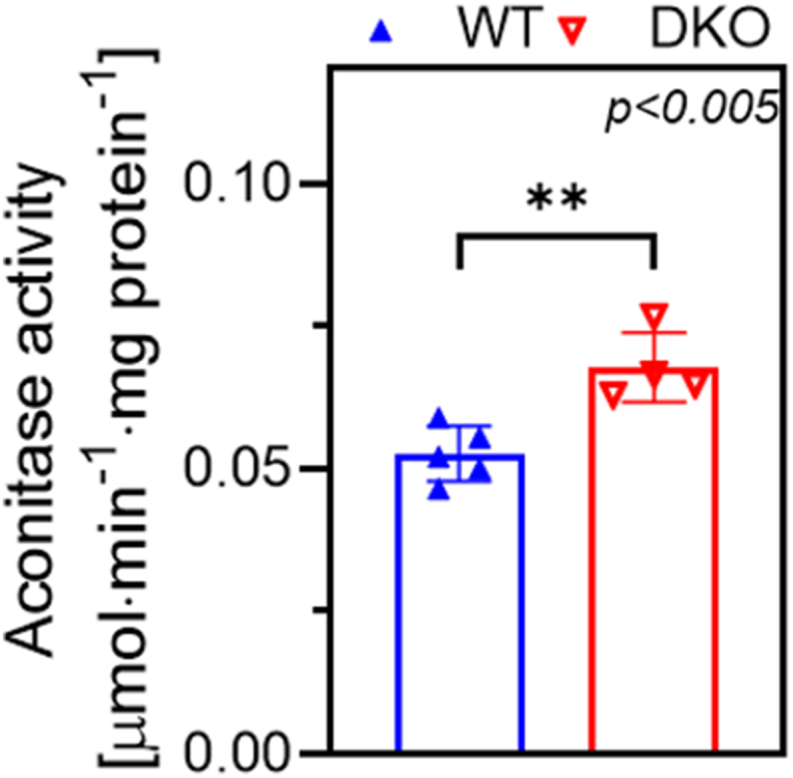
**Aconitase activity in kidney mitochondria of the WT and DKO mice.** The enzymatic activities were measured after 15-min incubations with 10 mM succinate. The activity values were normalized to the citrate synthase activity. Data are represented as means ± S.E.M. Data were analyzed by the unpaired *t*-test; n = 9/group. Further experimental details can be found in the Materials and Methods section.

Aconitase is the most sensitive enzyme to H_2_O_2_ in the TCA cycle [19,92]; its compromised activity generally indicates accelerated H_2_O_2_ formation in the mitochondrion (a.k.a. mitochondrial oxidative stress). Before measuring aconitase activity, kidney mitochondria were incubated with 10 mM succinate for 15 min at 37 °C to simulate RET *in vitro* (Fig. 5). In the DKO kidney mitochondria, the aconitase activity increased by 128 ± 7 % (0.068 ± 0.003 μmol ·min^−1^·mg protein^−1^) relative to control (0.053 ± 0.002 μmol ·min^−1^·mg protein^−1^); this result is in accord with the declined succinate-induced H_2_O_2_ production observed in the DKO brain mitochondria (Fig. 4b–d).

Correlating to our earlier studies [17,41] as well as others [15,16], the majority of ROS is produced by E3 subunits under high NADH/NAD^+^ ratio. Consequently, knocking out E2k does not provide additional protection against ROS.

5 Compensation in the TCA cycle

Since the KGDHc is a heavily regulated enzyme in the TCA cycle, we anticipated that its deficiency might trigger compensatory upregulation of certain TCA cycle-related enzymes. Surprisingly, the mRNA levels of two anaplerotic enzymes, namely glutamate dehydrogenase (glutamate + NAD^+^ ↔ α-ketoglutarate + NADH + H^+^; EC 1.4.1.2) and pyruvate carboxylase (pyruvate + CO_2_ + ATP ↔ oxalacetate + ADP + P_i_; EC 6.4.1.1), were unaltered in the DKO mouse liver relative to control (Fig. S3a and S3b). Along these lines, the mRNA level of succinate dehydrogenase (succinate + FAD^+^ ↔ fumarate + FADH_2_; EC 7.1.1.12), which operates downstream of the KGDHc catalyzed reaction in the TCA cycle, also remained unaltered in the DKO mouse liver relative to control (Fig. S3c). Furthermore, the mRNA level of the cytosolic ATP:citrate lyase (ATP + citrate + CoA + H_2_O→oxalacetate + acetyl-CoA + ADP + P_i_; EC 2.3.3.8) was also unaltered; this shows that fatty acid synthesis *via* citrate was not intensified in the DKO mice relative to control (Fig. S3d).

In summary, the heterozygous knockout of both the E2k and E3 subunits in the KGDHc leads to significant decline in the overall mitochondrial function as evidenced by the substrate-specific decrease in the ATP synthesis, O_2_ flux and RET-induced or α-KG and rotenone-initiated H_2_O_2_ production. This raises the question whether these *in situ* alterations are reflected in measurable phenotypic and *in vivo* metabolic changes.

6 Body composition, metabolic parameters, and amino acid/acylcarnitine profiles in DKO mice

We anticipated that a TCA cycle deficiency might affect the body composition and overall mass of the animals due perhaps to altered nutrient utilization. Surprisingly, the age-matched DKO and WT animals displayed no significant differences in body, lean, and fat masses, suggesting the lack of an *in vivo* metabolic compensation under resting, physiological condition (Fig. S4). The mice were also placed in metabolic cages to measure their daily food intake, horizontal and vertical activities, and respiratory exchange ratios (RER); RER is calculated from the ratio of exhaled CO_2_ and inhaled O_2_ (Fig. S5). Interestingly, none of these parameters showed significant differences between the genotypes at room temperature and later in thermoneutral cages (at 30–31 °C). These results rule out that the *DLST* and/or *DLD* genes would have any role in thermoregulation.

The proper degradation of most amino acids is connected to a functional TCA cycle or an α-keto acid dehydrogenase complex. Therefore, we analyzed the amino acid profiles in our animals using sera (Fig. S6) and DBS (Fig. S7) samples. The data showed no differences in the amino acid profiles tested between the DKO and WT groups. In particular, the branched-chain amino acids (valine, isoleucine, leucine), which require the BCKDHc for their oxidative decarboxylation, glycine (the substrate of the E3-containing GCS), lysine and tryptophan (which require the KADHc for their oxidative breakdown), did not significantly differ between the DKO and WT samples.

The acylcarnitine profiles of these mice were also analyzed from DBS samples to assess the mitochondrial fatty acid oxidation and utilization (Fig. S8): No (short, medium or long-chain) acylcarnitines differed between the DKO and control animals. This suggests that (*1*) the mitochondrial beta-oxidation pathway was unaffected, (*2*) the fatty acid transport into the mitochondrion (*via* acylcarnitines) was not disturbed, and (*3*) fatty acid utilization is not predominant in the DKO animals.

7 Treadmill fatigue-endurance test for DKO animals

**Fig. 6:**
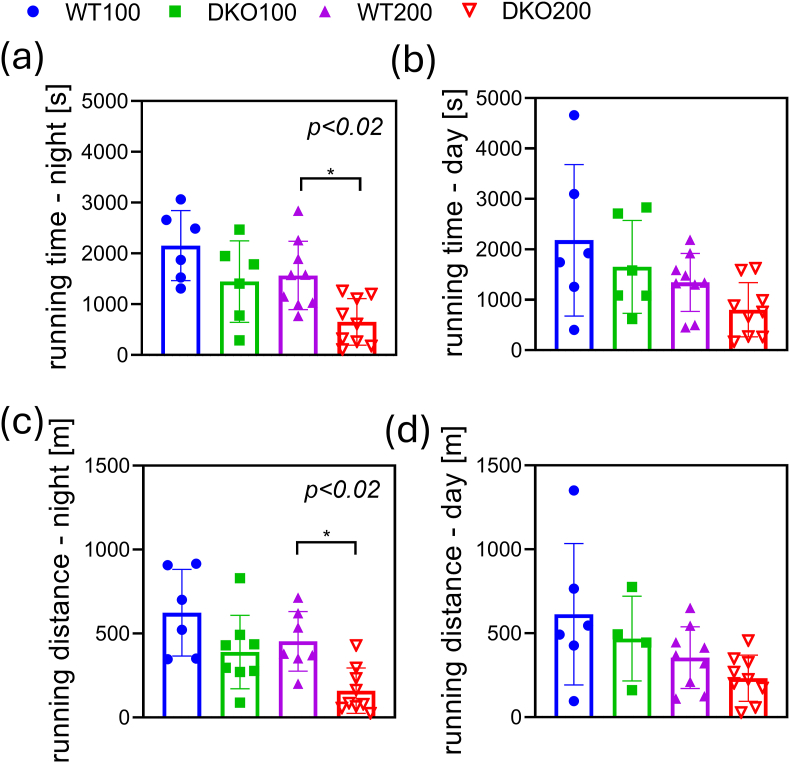
**Running time and distance in the treadmill fatigue test for DKO and WT mice at night and daylight.** After two days of conditioning, the mice were forced to run until full exhaustion. The running time and distance of the DKO200 mice significantly declined as compared to WT. Data are expressed as means ± S.E.M. Data were analyzed by two-way ANOVA followed by Sidak's multiple comparisons test; n = 6–11/group.

As was observed in prior experiments, the mice metabolically compensate well *in vivo* for a heterozygous double-knockout affecting the TCA cycle under normal, resting circumstances. The question arose as to how the DKO mice would behave under stress. The treadmill fatigue test was performed until full exhaustion on a motor-driven treadmill with increasing velocity [71]. The running distance and time both decreased by 64.8 and 63.9 %, respectively, in the DKO200 mice during the active phase, at night (Fig. 6a–c). These data suggest that in KGDHc deficiency in the course of an intense (muscle) workout the energy demands can be less satisfied due to reduced mitochondrial ATP synthesis and O_2_ expenditure. During the inactive phase, the DKO200 mice appeared to perform the worst as compared to the controls (Fig. 6b–d). Similarly, the running performance of the younger DKO animals was not significantly reduced relative to the controls. Altogether, these data suggest that the middle-aged DKO mice were less capable of metabolically compensating than the younger DKO animals for KGDHc deficiency.

**Fig. 7:**
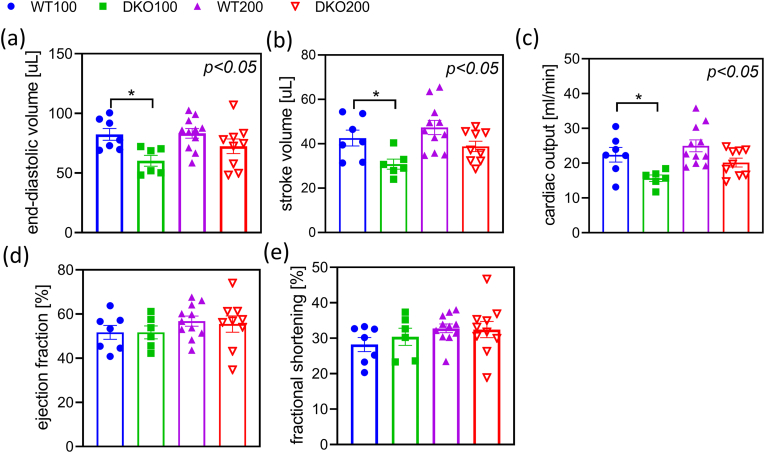
**Echocardiographic measurements for cardiac output in DKO animals.** The end-diastolic volume (a) and stroke volume (b) both reduced in the DKO animals that led to a lower cardiac output (c). The ejection fraction (d) and fractional shortening (e) were maintained. Data are expressed as means ± SEM. Data were analyzed by two-way ANOVA followed by Sidak's multiple comparison's test; n = 5–10/group.

We hypothesized that cardiac dysfunction might be a reason for faster exhaustion in the forced treadmill test. Therefore, echocardiography was performed (Fig. 7 and S9). We found that the LVEDV and SV values decreased in the DKO animals, which resulted in a significant decrease in the CO value, as well. However, the EF and FS values remained unchanged. The diastolic functions as measured *via* the E to e’ ratio and IVRT value were altered. No major cardiac remodeling could be observed *via* estimating the LV mass and calculating the LVRI value. Based on the above data, we suppose that the DKO mice possess mildly compromised resting cardiac functions that likely worsen upon physical activity; this would corroborate the reduced performance in the endurance test.

We also hypothesized that fibrotic alterations in the skeletal muscles might have also contributed to the compromised endurance of the DKO mice. To investigate this, we applied Sirius red staining to assess the fibrosis levels in the skeletal muscle samples (Fig. S10). With examining at least four samples in each animal group, we observed no fibrotic changes in the DKO mice. These findings suggest that the decline in performance is due likely to metabolic alterations and deteriorating cardiac activity/nutrient utilization. Based on these results, we propose that the DKO mice possess weaker compensatory efficacy under stress conditions, like forced workout.

8 Locomotor activities of the DKO mice in an open-field test

**Fig. 8:**
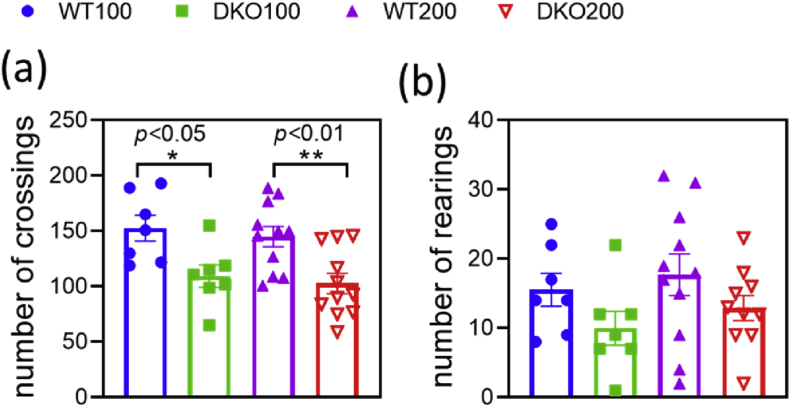
**Novelty-induced locomotion activity by DKO mice in the open-field test.** The number of crossings (horizontal activity) (**a**) and rearings (vertical movement) (**b**) in the whole arena are reported in the four different animal groups. Data are expressed as means ± S.E.M. Data were analyzed by two-way ANOVA followed by Sidak's multiple comparison test; n = 6–11/group.

KGDHc deficiency is often associated with cognitive decline and reduced motor functions; for review, see: [25]. However, the relevant literature reports no behavioral tests on respective knockout animals. To investigate whether compromised KGDHc activity can indeed lead to impairments in motor and cognitive functions, DKO and WT mice underwent various behavioral and memory tests. In the open-field test, the novelty-induced horizontal activity (number of crossings) significantly decreased in both the DKO100 and DKO200 groups as compared to the controls (Fig. 8a). The vertical activity (number of rearings) also showed a declining trend in the DKO groups relative to control (Fig. 8b). However, the time periods spent in the inner and outer zones were similar in both genotypes (Fig. S11). The Morris water maze test and the novel object recognition test were conducted only with the middle-aged DKO mice to test their cognitive functions. The working memory was markedly worse in the DKO animals as compared to controls, whereas any difference in the reference memory was only presumable (Fig. S12). The recognition index did not differ between the genotypes, although the exploration time tended to decrease in the DKO group (Fig. S13). Overall, decline in both the vertical and horizontal activities in the open-field test indicated reduced motor functions and coordination in the DKO mice. Along this line, the DKO mice required considerably more time to reach the platform in the Morris water maze test, further suggesting impaired motor functions. These results indicated a minor cognitive decline in the middle-aged DKO mice. Previous studies reported compromised KGDHc activities in the post-mortem brains of AD patients, correlating well with dementia [93]. Decline in the KGDHc activity was also linked to memory loss and inefficient neurogenesis in animal models [44,93,94].

9 Microgliosis and neuronal cell death in the DKO mouse brains

**Fig. 9:**
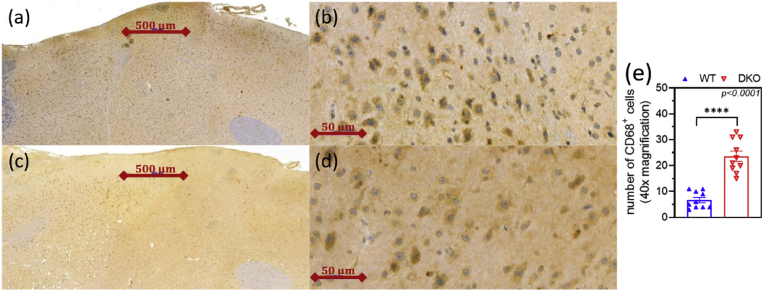
**CD68^+^ immunostaining of the cerebral cortex.** The cortices of the DKO animals (**a**, **b**) exhibited massive microgliosis relative to controls (**c**,**d**). The CD68^+^ cell counting was performed in 40x magnification and at ten different points in the cortex/group; n = 3–4 animals/group (**e**). Data are shown as means ± S.E.M. and were analyzed by the unpaired *t*-test.

To determine whether the changes observed in the behavioral tests are due to structural modifications or individual variability, histological samples were taken from the brains. CD68 (or macrosialin in rodents) is a lysosome-associated membrane protein (a.k.a. LAMP) primarily expressed in microglia in the brain [95]. The CD68 antigen is upregulated in pro-inflammatory cytokine-activated macrophage-like cells (e.g. microglia) but not in resting cells [96]. Microgliosis indicates that cortical neurons are damaged due to energy shortage, leading to the appearance of damage-associated molecular patterns (known as DAMPs) that activate microglia [97,98]. In the cerebral cortex of the DKO mice, substantial microgliosis (WT200: 6.7 ± 1, DKO200: 23.6 ± 2 CD68^+^ positive cells/visual field, at 40x magnification) was found through anti-CD68^+^ immunostaining (Fig. 9e), pointing to neuroinflammation [99,100]. An earlier study highlighted that brain samples from *DLST*^+/−^ or *DLD*^+/−^ mice displayed signs of disturbed neurogenesis, but no further abnormalities [94]. This is the first study to report microgliosis in KGDHc knockout animals, underscoring the consequences of cumulative genetic alterations.

To better understand the *in vivo* changes observed in behavioral tests, immunohistology samples from the hippocampal region were prepared using the terminal deoxynucleotidyl transferase-mediated dUTP-biotin nick end labeling (TUNEL) method. In this staining procedure, the terminal deoxynucleotidyl transferase specifically binds to the free 3′-OH end of DNA [101]. Consequently, fragmented DNA results in more binding sites, increasing the staining intensity. In the older DKO group, the CA1 area of the hippocampus displayed massive DNA fragmentation as compared to the age-matched controls (Fig. S14). The CA1 subfield is a higher-level comparator in the hippocampus, receiving input from CA3 and also the entorhinal cortex [102,103]. Malfunctions in this area could lead to behavioral changes in the animals. Additionally, several earlier studies reported that even mild cognitive impairment can be accompanied by CA1 atrophy [104,105]. Notably, younger animals are expected to better tolerate intense proinflammatory signals, as described by other researchers [106]. Importantly, DKO murines exhibited significant signs of neurological dysfunction with applying no neurotoxins or exogenous H_2_O_2_, unlike it was previously observed in single heterozygous *DLST*^+/−^ mice [44,107].

**Fig. 10:**
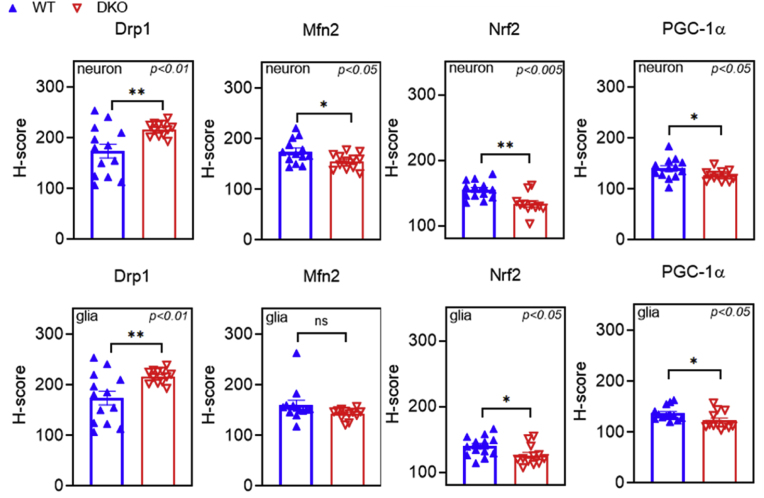
**Expression levels of dynamin-related protein 1 (Drp1), mitofusin 2 (Mfn2), nuclear factor (erythroid-derived 2)-like 2 (Nrf2) and peroxisome proliferator-activated receptor gamma coactivator 1-alpha (PGC-1α) in neurons and glial cells in the wild-type and double-knockout mice.** Drp1 was elevated in both cell types, whereas Mfn2 decreased in neurons referring to a more intense mitochondrial fission in the DKO mice. Nrf2 and PGC-1α, reduced in both cell types in the mutant animals. H-score refers to relative expression level and was calculated as described under Materials and Methods. Representative illustrations are shown in Fig. S15. Data are expressed as means ± S.E.M. and were analyzed by the unpaired *t*-test; n = 11–12/group.

Hippocampal neuronal loss and alterations in mitochondrial dynamics were observed in the course of cognitive decline and in select neurodegenerative diseases [[108], [109], [110]]; for review see: [111]. In both the neurons and glial cells in the DKO animals, the relative expression of the dynamin-related protein 1 (Drp1), a regulator of mitochondrial fission, was elevated (Fig. 10). Correspondingly, the relative expression of mitofusin 2 (Mfn2), which plays a central role in mitochondrial fusion, reduced in the DKO mice (Fig. 10). These findings align with previous studies [108,110] that reported elevated Drp1 and decreased Mfn2 levels in the cortices of AD patients and in SH-SY5Y neurons with reduced KGDHc activity. Consequently, increased mitochondrial fragmentation and altered mitochondrial dynamics were observed in these models. The mitochondrial transcription factor A (MTFA) levels did not change in the knockout groups relative to controls, indicating that these mutations do not affect or modify the mitochondrial DNA [112] (Fig. S16). Frank and co-workers reported that the level of Drp1 increased in response to mild stress with no considerable autophagy or cell death [113]. Taken together with our results, we can conclude that neuronally derived cells effectively compensated for a long time through enhanced mitochondrial fission, hence only older animals showed significant deviations from the control group.

To maintain this state, Mfn2 expression must be reduced, allowing well-functioning mitochondria to overcome the heterozygous mutation of the key enzyme in the TCA cycle. According to our hypothesis “underperforming” mitochondria may be consumed by autophagolysosomes, while “overperforming” mitochondria remain intact and attempt to compensate for the heterozygous genetic modification. This mechanism may explain why we do not observe one-half decline in our *in vivo* results. Our present findings point out that this compensatory mechanism may be effective only for a limited time and under mild stress (e.g. treatment of a cell line for a few hours). In the case of heterozygous knockout of both *DLST* and *DLD* genes, the persistent energy production deficiency may result in a life-long mild stress, eventually leading to cognitive symptoms. These symptoms could not be detected in single KO animals without neurotoxical treatment [44], as the threshold for pathological manifestations is higher in the latter rodents.

The regulator of mitochondrial biogenesis, the peroxisome proliferator-activated receptor gamma coactivator 1-alpha (PGC-1α), and the nuclear factor (erythroid-derived 2)-like 2 (Nrf2, also called as NFE2L2), both decreased in concentration in both the neurons and glial cells in the DKO mice as compared to the WT animals (Fig. 10). Nrf2 is not only regulated by the well-known Keap1-Nrf2 pathway [114], but Aquilano and colleagues also found that PGC-1α and Nrf2 are parts of the same redox-signaling pathway controlled by p53 [115]. Previous studies reported that reduction in the PGC-1α level in mitochondrial impairment is often associated with inflammation, neurite damage, and cell death [[116], [117], [118], [119]]. Studies on PGC-1α and Nrf2 knockout mice point out an age-dependent worsening in macular degeneration ([120]; Sridevi [121]). Additionally, decreased Nrf2 expression was correlated with cognitive decline and AD-like symptoms [122]. In various mouse models of AD, both the altered mitochondrial dynamics and decreased PGC-1α levels have been linked to mitochondrial dysfunction, leading to cognitive decline and disease manifestation [123,124]. Based on our data, KGDHc affects PGC-1α-related pathways, hereby downregulation of the enzyme negatively affects mitochondrial biogenesis and stress response answers (e.g. Nrf2). Additionally, Nrf2 can also be activated by oxidative stress (for review see: [125,126]. Therefore, the measured Nrf2 concentration reflects both KGDHc dysfunction and mild oxidative stress, as indicated by the decreased Drp1 concentration (Fig. 10). This means that in DKO mice the reduced Nrf2 expression is mostly affected by the consequences of KGDHc downregulation; the effect of oxidative stress is negligible.

Altogether, these results show that reduced overall KGDHc activity in the DKO animals is associated with minor cognitive decline. These findings are further supported by several other observations, including microgliosis, increased hippocampal neuronal death, and altered mitochondrial dynamics and biogenesis. These central nervous system changes may not be directly related to mitochondrial ATP synthesis efficiency but could indeed be linked to altered glutamate metabolism. Reduced overall KGDHc activity can lead to increased glutamate formation *via* enhanced GDH activity in neurons, resulting in glutamate toxicity and neuronal death [127,128]. In the DKO mice, DAMPs released by dead neuronal cells may have triggered neuroinflammation, further exacerbating neuronal death and contributing to cognitive decline [129,130]. Additionally, the DKO did not affect the daytime activity, but significantly altered the nighttime activity, which is primarily used for exploration and social interactions, suggesting the presence of neuronal disorder. This is also evident from the impaired novelty-induced locomotion activity in the open-field test. However, the question arose as to why the *in vivo* results are not as robust as the immunohistology images. The most plausible explanation is that compensatory mechanisms at the whole-body level could in some way effectively counterbalance the genetic alterations. The question arises: Would using senescent mice or creating a genetic crossover with known AD-related model result in a better model with a more afflicted phenotype?

10 Glucose uptake capacity analysis in the DKO mouse brains using PET-CET imaging

In the context of the PET imaging analysis, we evaluated the standardized uptake values (SUV) of ^18^F-fluorodeoxyglucose (FDG) to assess glucose metabolism in the WT and DKO mice [131,132]. Since glucose is the major fuel for the brain, its catabolic rate can be correlated with neuronal function, neuroinflammation and synaptic density. After imaging acquisition, we delineated regions of interest (ROIs) based on CT scans for the brain and heart (Fig. S17). The SUV of FDG was higher in both organs in the DKO group as compared to the controls, approaching significance. The question arises as to what explains the increased glucose utilization in organs experiencing energy deficits due to KGDHc dysfunction. On one hand, glucose hypermetabolism could be a route of metabolic compensation in the DKO brain cells (mostly neurons) and cardiomyocytes for a less efficient TCA cycle and insufficient OXPHOS. On the other hand, as shown in Fig. 9, neuronal dysfunction was accompanied by the accumulation of inflammatory cells such as microglia (microgliosis), which express glucose transporters. Thus, microglia could indeed contribute to the increased glucose uptake and altered brain metabolism [133,134]. These findings align well with the observed minor cognitive decline and the deterioration of cardiac function in the DKO mice.

## 4 Conclusions

This study demonstrates that the double-heterozygous knockout of the E2k and E3 subunits of the KGDHc led to significant substrate-specific alterations in mitochondrial bioenergetics *in vitro*, but only minor metabolic and behavioral differences were observed between the two genotypes *in vivo* under physiological, resting conditions. The major finding of our study is that the middle-aged transgenic mice performed significantly poorer than the corresponding younger animals under high energy demand situations, like stress conditions. Under these circumstances, the functional alleles of the *DLST* and *DLD* genes were incapable of counterbalancing the altered ATP utilization. In the fatigue-endurance test, a running workout, the DKO animals got exhausted significantly faster and were capable of running to an overall shorter distance when compared to their aged-matched controls. This could be explained not only by the energy deficit, but also the mildly decreased resting cardiac function, which worsened upon activity. Additionally, we observed that the heterozygous knockout of both *DLST* and *DLD* leads to microgliosis in the cerebral cortex, neuronal death in the hippocampus, alterations in the mitochondrial dynamics and biogenesis. These findings indicate neuronal involvement and suggest minor cognitive decline, which was also in part reflected in the behavioral tests. These data are in accord with the literature [135], unequivocally demonstrating that the heterozygous malfunction of the KGDHc is often not noticeable in a healthy, physiological state, but it disturbs the adaptation and compensatory responses of the system in disease states or stress situations, manifesting in pathophysiological symptoms. Many homozygous human mutations of KGDHc are accompanied by severe, life-threatening symptoms, often resulting in early childhood death. Therefore, future studies on relevant human homozygous KGDHc mutations - such as E375K in hE3 - using cell lines and rodent models would be valuable for elucidating the biochemical pathomechanism of these diseases and developing potential treatment strategies.

## CRediT authorship contribution statement

**Márton Kokas:** Formal analysis, Investigation, Methodology, Visualization, Writing – original draft, Writing – review & editing. **András Budai:** Formal analysis, Investigation, Methodology. **Andrea Kádár:** Formal analysis, Investigation, Methodology. **Soroosh Mozaffaritabar:** Formal analysis, Investigation. **Lei Zhou:** Formal analysis, Investigation. **Tímea Téglás:** Methodology. **Rebeka Sára Orova:** Formal analysis, Investigation. **Dániel Gáspár:** Formal analysis, Investigation. **Kristóf Németh:** Formal analysis, Investigation. **Daniel Marton Toth:** Formal analysis, Investigation, Methodology. **Nabil V. Sayour:** Formal analysis, Investigation, Methodology. **Csenger Kovácsházi:** Formal analysis, Investigation, Methodology. **Andrea Xue:** Formal analysis, Investigation, Methodology. **Réka Zsuzsanna Szatmári:** Formal analysis, Investigation, Methodology. **Beáta Törőcsik:** Formal analysis, Investigation, Methodology. **Domokos Máthé:** Formal analysis, Investigation, Methodology. **Noémi Kovács:** Formal analysis, Investigation, Methodology. **Krisztián Szigeti:** Conceptualization, Resources, Writing – review & editing. **Péter Nagy:** Conceptualization, Resources, Writing – review & editing. **Ildikó Szatmári:** Conceptualization, Resources, Writing – review & editing. **Csaba Fekete:** Conceptualization, Resources, Writing – review & editing. **Tamás Arányi:** Conceptualization, Resources, Writing – review & editing. **Zoltán V. Varga:** Conceptualization, Resources, Writing – review & editing. **Péter Ferdinandy:** Conceptualization, Resources, Writing – review & editing. **Zsolt Radák:** Conceptualization, Resources, Writing – review & editing. **Andrey V. Kozlov:** Funding acquisition, Resources, Writing – review & editing. **László Tretter:** Conceptualization, Supervision, Writing – review & editing. **Tímea Komlódi:** Conceptualization, Formal analysis, Investigation, Methodology, Project administration, Visualization, Writing – original draft, Writing – review & editing. **Attila Ambrus:** Funding acquisition, Resources, Writing – review & editing.

## Declaration of competing interest

Peter Ferdinandy is the founder and CEO of the Pharmahungary Group, a group of R&D companies. All other authors declare no conflict of interest.
