# Supplementary material for: Microgliosis, neuronal death, minor behavioral abnormalities and reduced endurance performance in alpha-ketoglutarate dehydrogenase complex deficient mice
Source: Redox Biol. 2025 Jun 27;85:103743. doi: 10.1016/j.redox.2025.103743 (PMC12271819; doi:10.1016/j.redox.2025.103743)
Supplement: Multimedia component 1 [file mmc1.docx]

**Supplementary material**

**Microgliosis, neuronal death, minor behavioral abnormalities and reduced endurance performance in alpha-ketoglutarate dehydrogenase complex deficient mice**

Kokas et al.

**Table S1. Oligonucleotide sequences for genotyping**

| Primer designation | Sequence | PCR product (bp) |
| --- | --- | --- |
| *Dld* wild type | CAT GGC TCC TTT CAG CTG TT | 340 |
| *Dld* common | ACA ATA TAC CCG CCT CAC CA |  |
| *Dld* mutant | TCG CCT TCT TGA CGA GTT CT | 232 |
| *Dlst* wild type | ATAAACCCTCTTGCAGTTGCATC | 186 |
| *Dlst* common | TAGGTTCCTAGGTAGGGATACAGC |  |
| *Dlst* mutant | CTACTCTCTAACCTACCAAGCTGG | 210 |

**
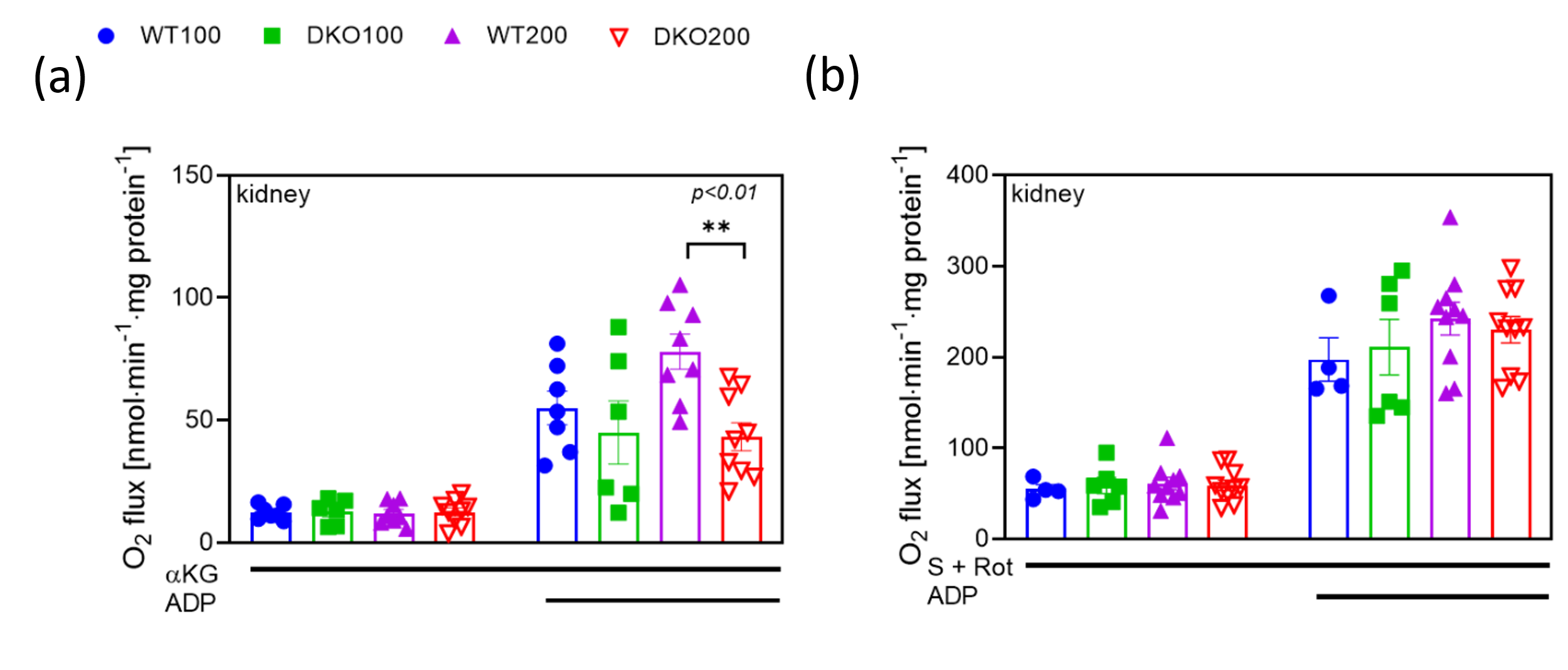
**

**Figure S1.** **O_2_ consumption in isolated kidney mitochondria energized with either α-KG (a) or succinate (b) in four different animal groups.** Representative traces of independent experiments are shown in Figure 1. Mitochondria (0.05 mg/mL) were incubated in the respiratory medium. α-KG or rotenone *plus* succinate, and afterwards ADP were added, as indicated. Data are represented as means±S.E.M. Data were analyzed by two-way ANOVA followed by Sidak’s multiple comparisions test; n=5-9/group. Lines under the panels indicate additions of chemicals into the respiratory medium and mitochondrial suspension. Further experimental details can be found in the Materials and Methods section.

**
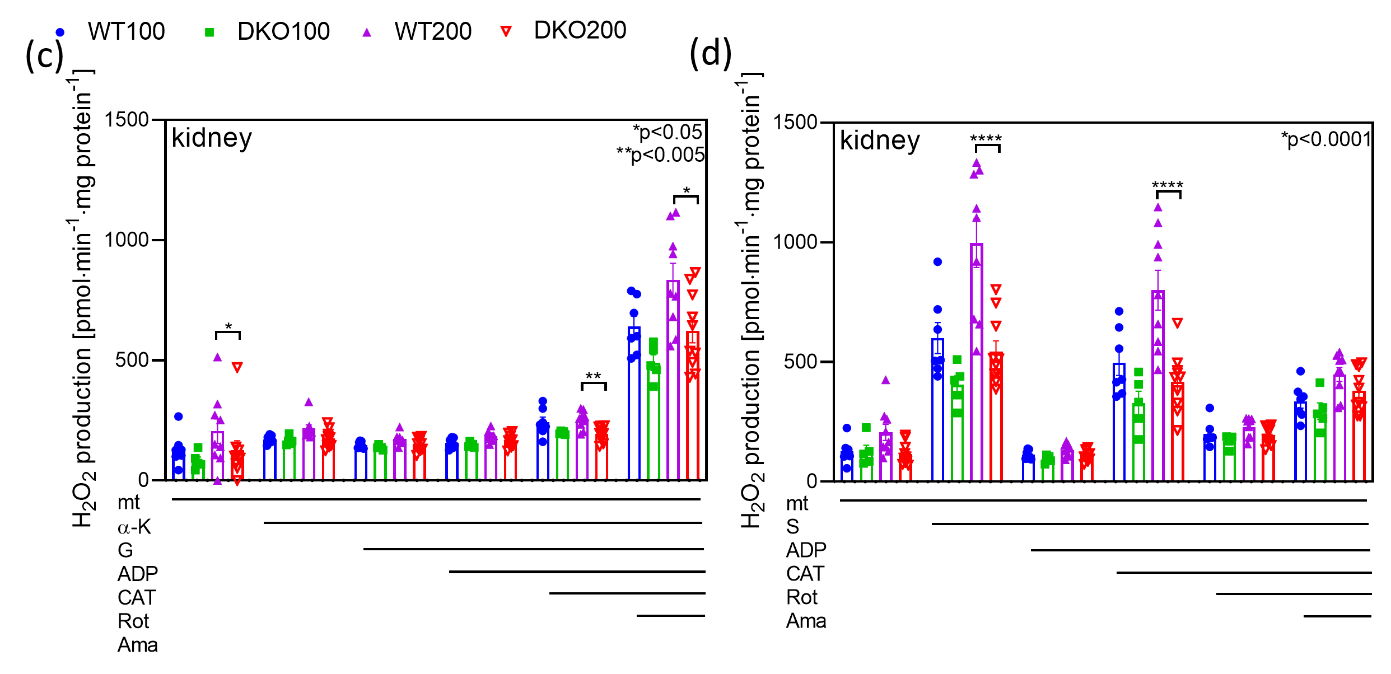
**

**Figure S2. H_2_O_2_ production in isolated kidney mitochondria energized with either α-KG (a) or succinate (b) in four different animal groups.** Representative traces of independent experiments are shown in Figures 4a and 4b. Mitochondria were administered into the respiratory medium after Amplex UltraRed and Horseradish peroxidase. Afterwards, α-KG or succinate, ADP, CAT, Rot, and Ama were given, as indicated. H_2_O_2_ production rates are expressed as means±S.E.M. Data were analyzed by two-way ANOVA followed by Sidak’s multiple comparisons test; n=4-9/group. Further experimental details can be found in the Materials and Methods section.

**
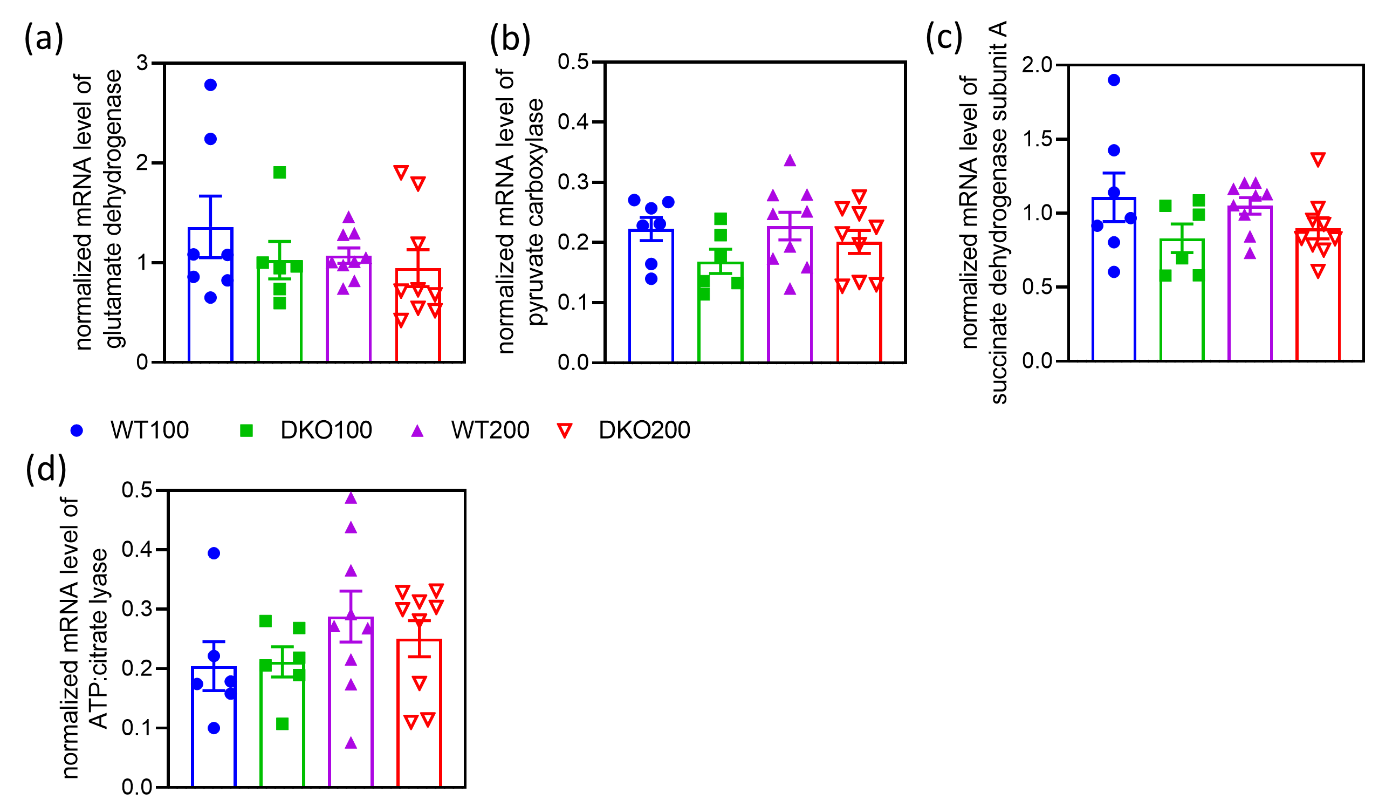
**

**Figure S3. Relative mRNA levels for the glutamate dehydrogenase (a), pyruvate carboxylase (b), succinate dehydrogenase subunit A (c), and ATP:citrate lyase (d).** The mRNA levels were normalized to ribosomal protein 29 (Rps29). Data are shown as means±S.E.M. Data were analyzed by two-way ANOVA followed by Sidak’s multiple comparisions test; n=5-9/groups. Experimental details can be found in the Materials and Methods section.

**
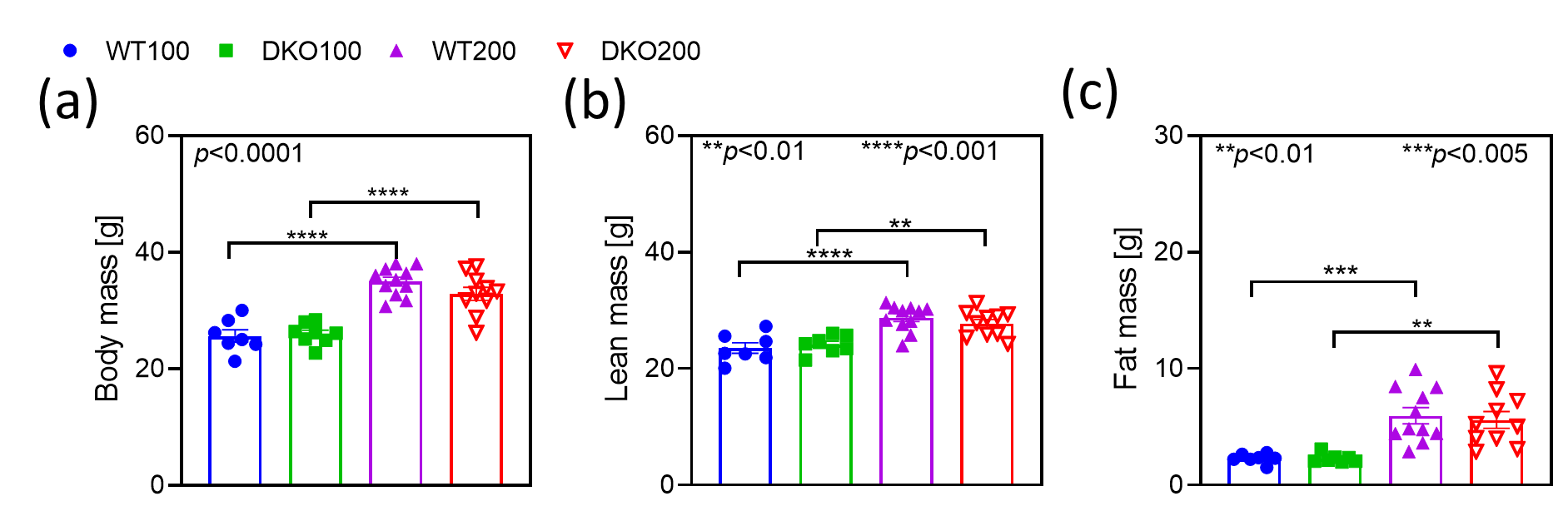
**

**Figure S4. Body mass (a) and body composition (b,c) in four different animal groups.** There were no significant differences between the DKO and their age-matched control animals. Data are expressed in grams (means±S.E.M.). Data were analyzed by two-way ANOVA followed by Sidak’s multiple comparisions test; n=6-11/groups. Experimental details can be found in the Materials and Methods section.


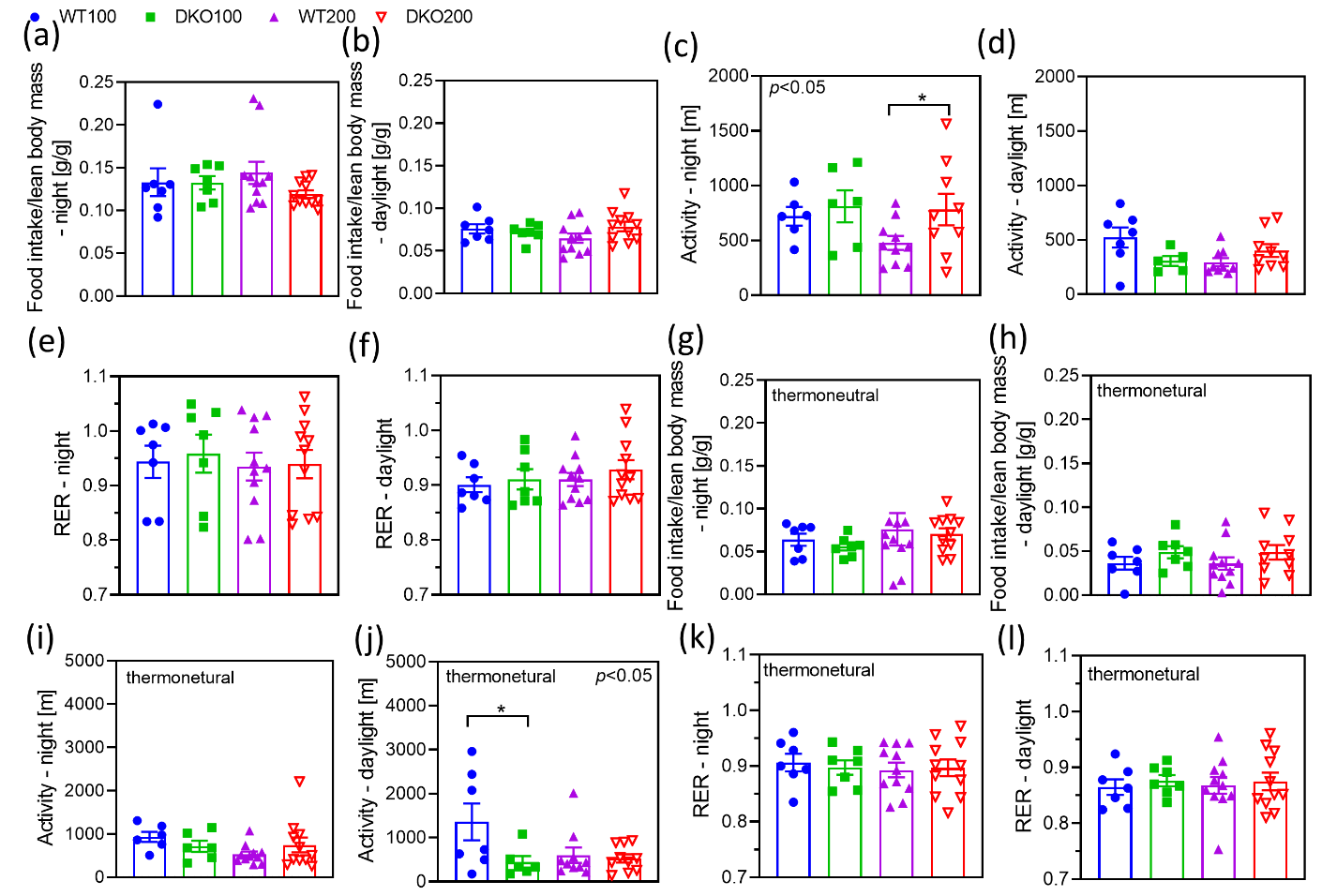


**Figure S5. Food intake, physical activity, and respiratory exchange ratio (RER) observed in single-animal metabolic cages for four different animal groups at room or thermoneutral temperatures combined with daylight or nighttime.** No significant differences could be observed between the corresponding DKO and control animals except for physical activity at nighttime in the middle-aged group. Data are shown as means±S.E.M. Data were analyzed by two-way ANOVA followed by Sidak’s multiple comparisions test; n=6-11/groups. Experimental details can be found in the Materials and Methods section.


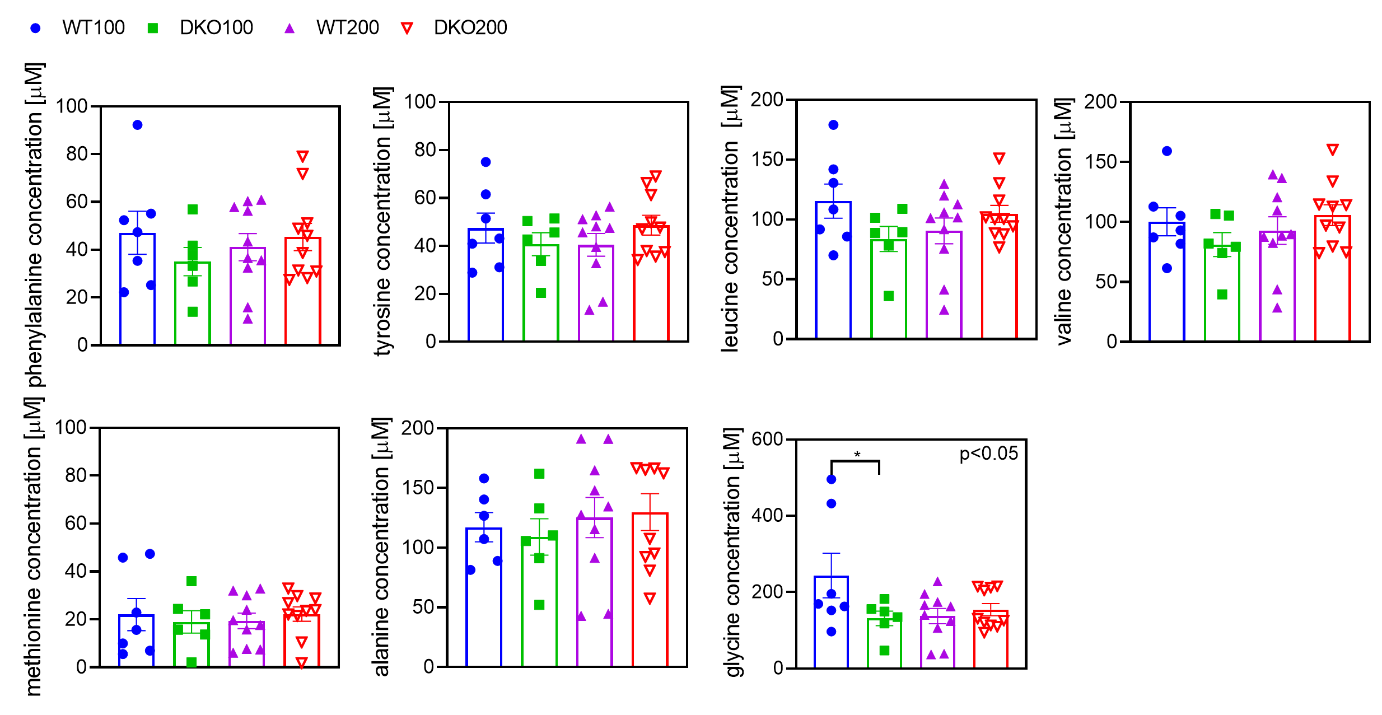


**Figure S6. Amino acid profiles in dried blood spots.** No significant differences could be detected between the corresponding DKO and WT animals. Data are expressed in µM (means±S.E.M.). Data were analyzed by two-way ANOVA followed by Sidak’s multiple comparisions; n=6-10/groups. Experimental details can be found in the Materials and Methods section.


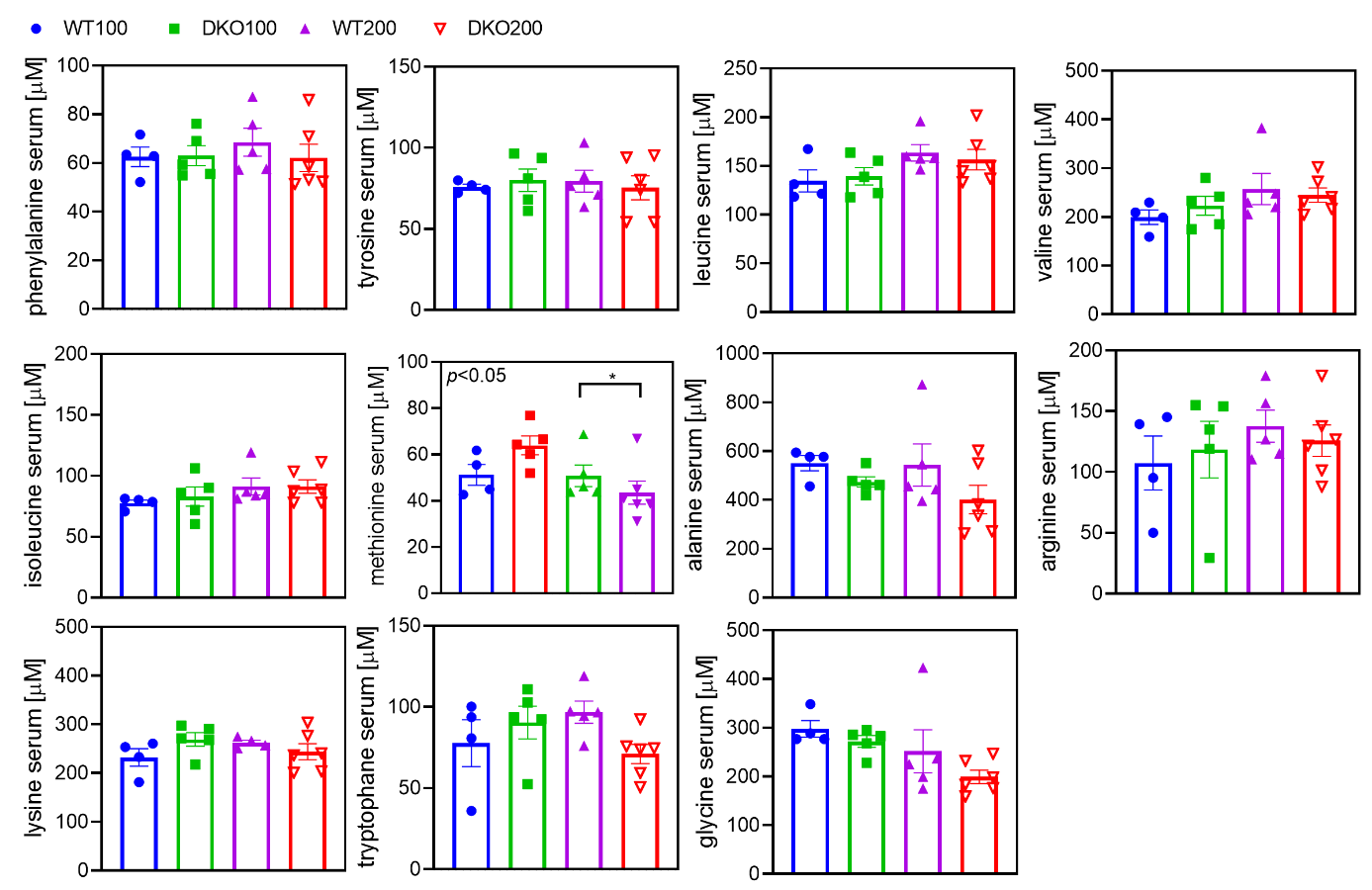


**Figure S7. Amino acid profiles in sera.** No significant differences could be observed between the corresponding DKO and WT animals. Data are expressed in µM (means±S.E.M.). Data were analyzed by two-way ANOVA followed by Sidak’s multiple comparisions; n=6-10/groups. Experimental details can be found in the Materials and Methods section.


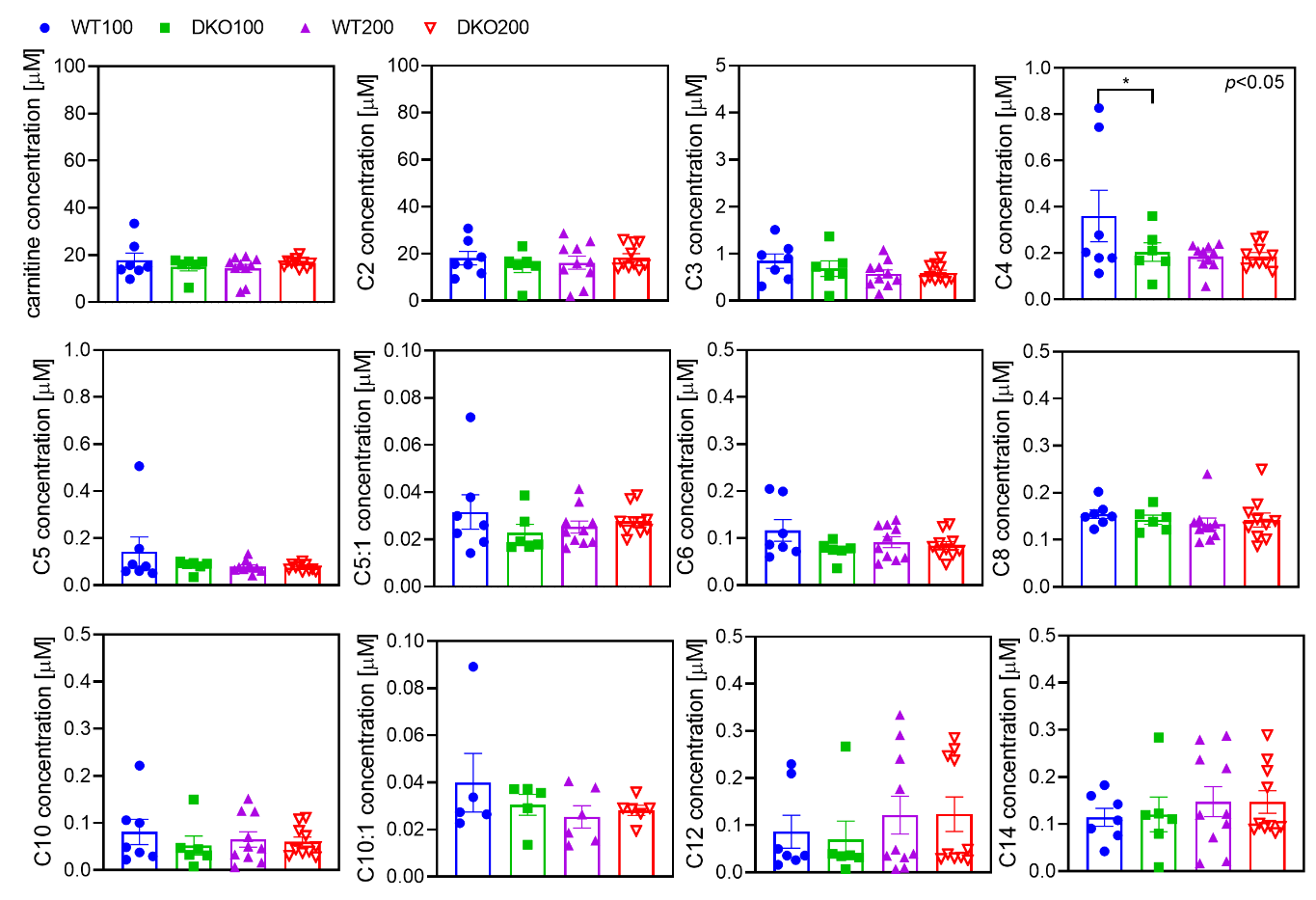


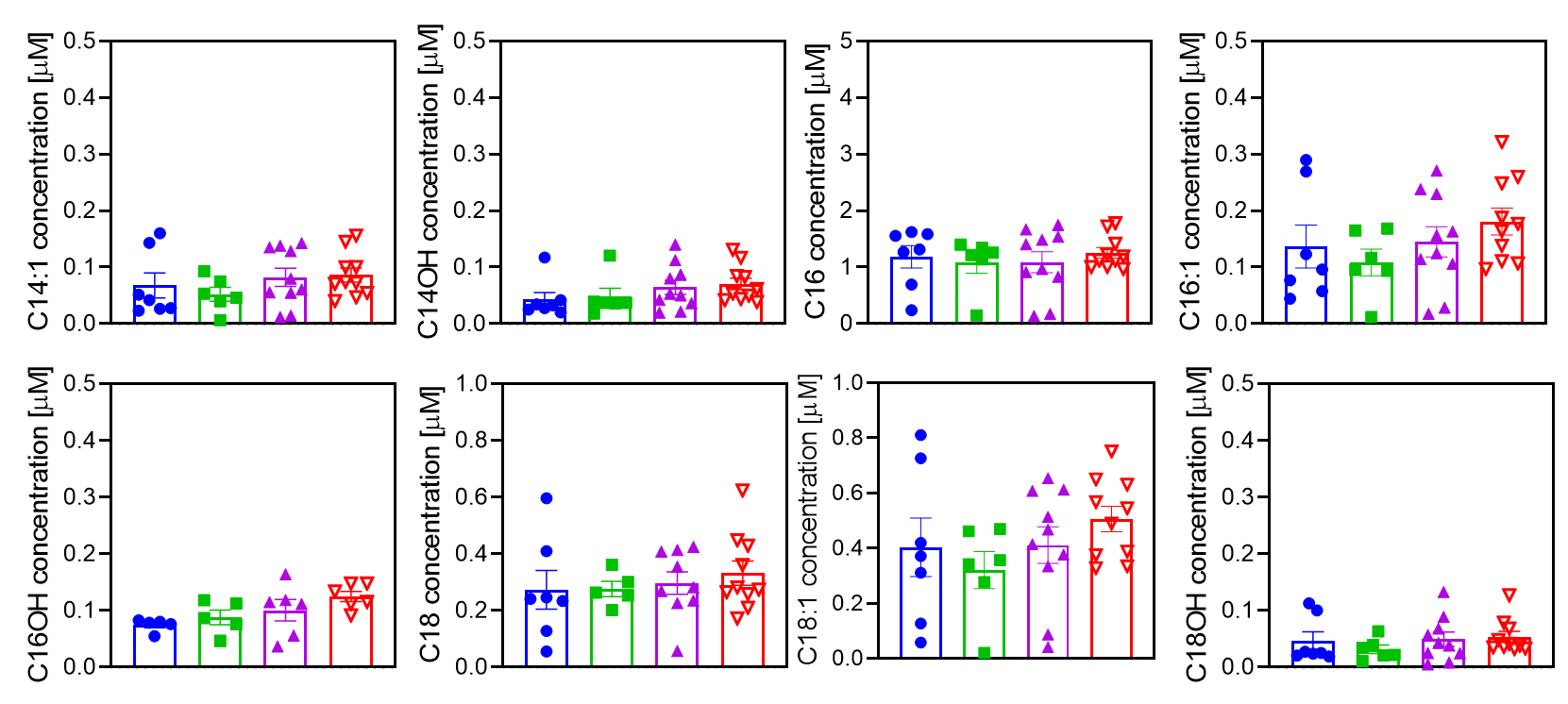


**Figure S8.** **Acylcarnitine profiles in dried blood spots.** No significant differences could be detected between the corresponding DKO and WT animals. „C” refers to the number of carbon atoms in the fatty acid in the respective acylcarnitine; e.g. C5:1 means a fatty acid with five carbon atoms and a single double bond (unsaturated fatty acid). „OH” refers to the hydroxylated form of the given fatty acid. Data are expressed in µM (means±S.E.M.). Data were analyzed by two-way ANOVA followed by Sidak’s multiple comparisons, n=5-10/group. Experimental details can be found in the Materials and Methods section.

**
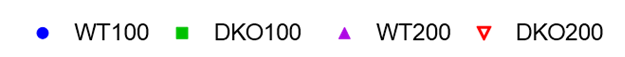
**


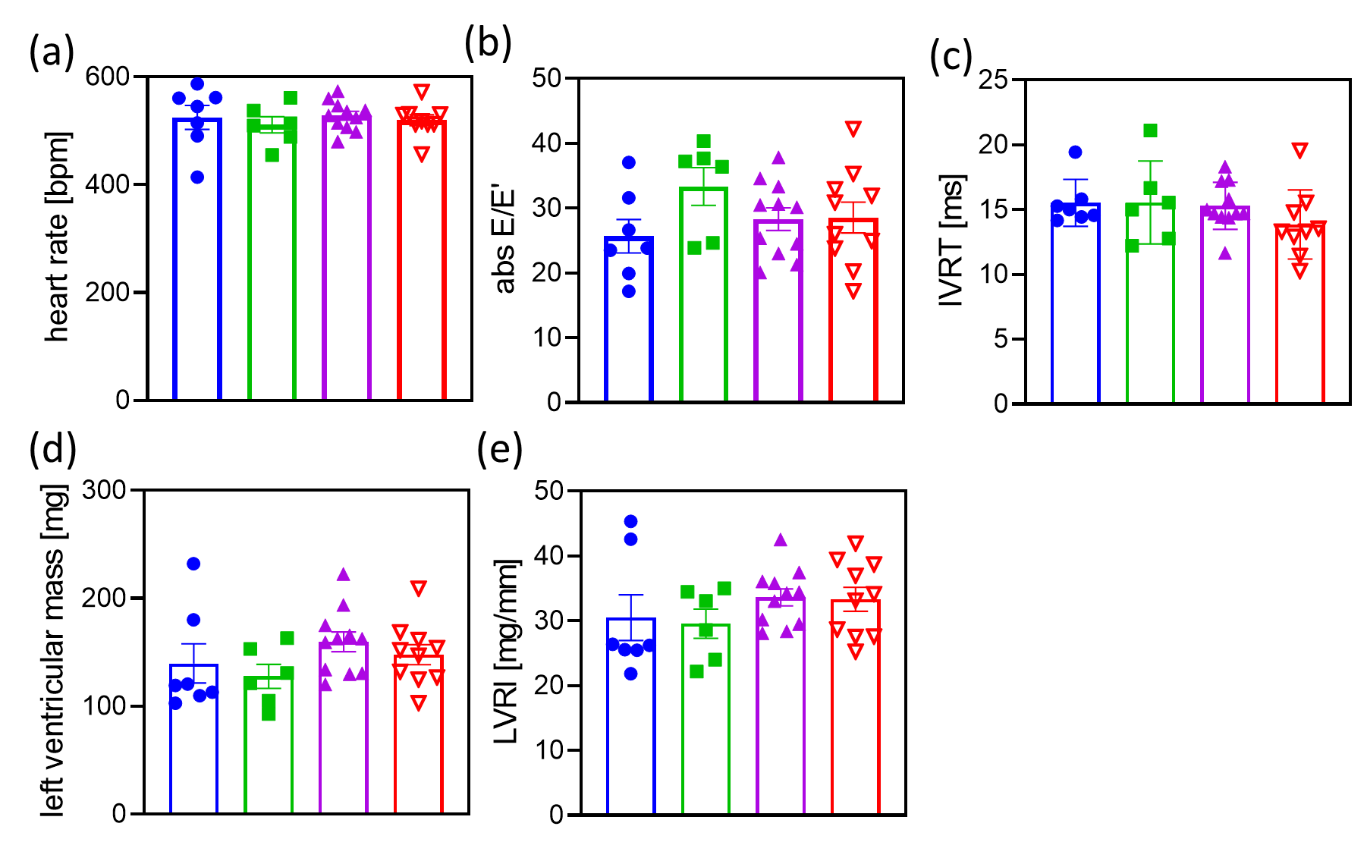


**Figures S9.** **Auxiliary** **echocardiographic measurements.** Heart rate (a), E to e’ ratio (b), isovolumetric relaxation time (IVRT) (c), estimated left ventricular mass (d), and calculated left ventricular remodeling index (LVRI) (e) were measured. No significant differences were observed in these parameters between the respective knockout and control groups. Data are expressed as means±S.E.M. Data were analyzed by two-way ANOVA followed by Sidak’s multiple comparisons; n=5-10/group. Experimental details can be found in the Materials and Methods section.

**
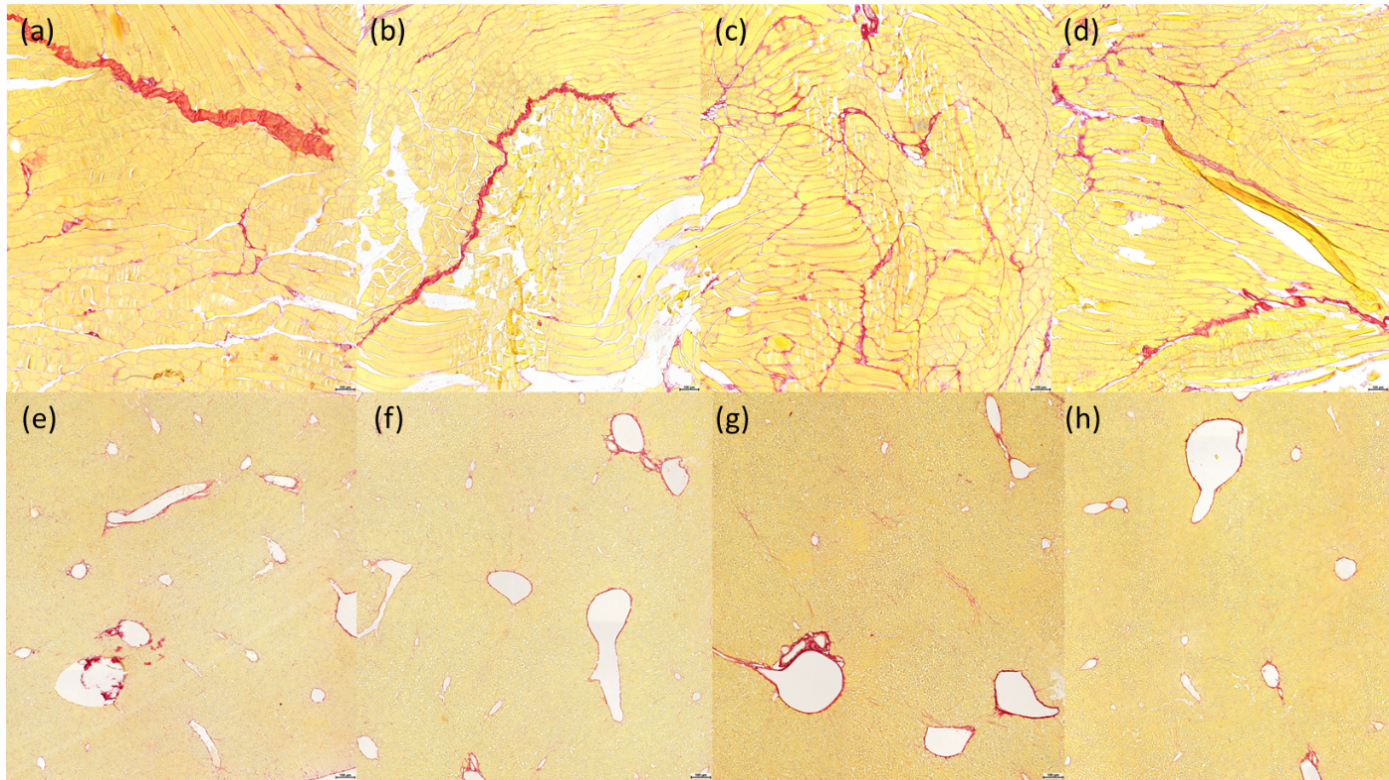
**

**Figure S10. Sirius red staining in mice skeletal muscle samples.** WT 100 (a), DKO 100 (b), WT 200 (c), DKO 200 (d). None of the samples displayed significant fibrotic alterations. Experimental details can be found in the Materials and Methods section.


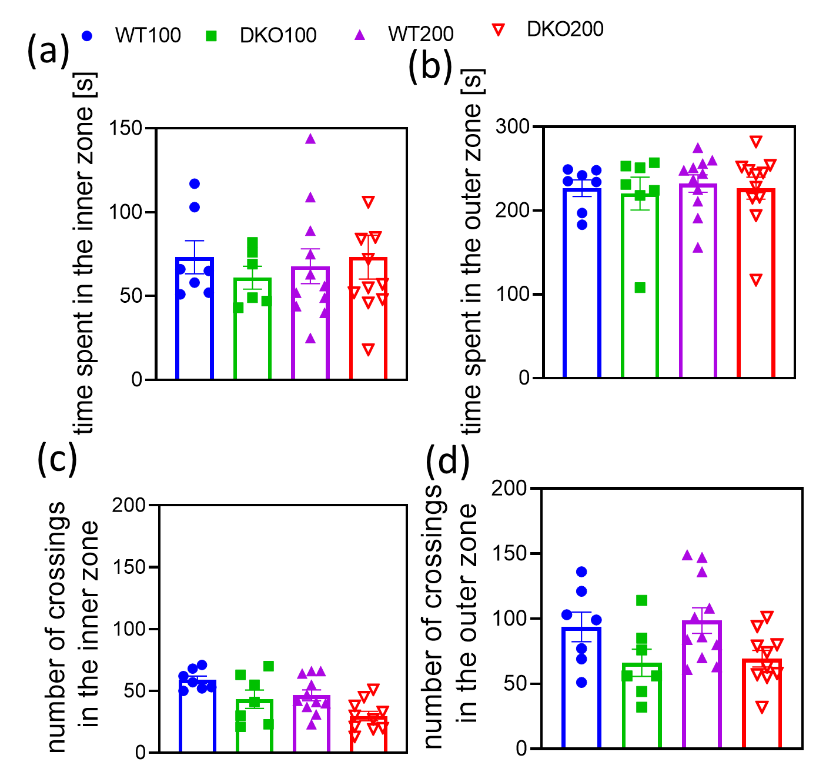


**Figure S11. Open field test: crossings and time-spans in the inner and outer zones.** The number of crossings (horizontal activity) refers to novelty-induced locomotion. Data are represented as means±S.E.M. Data were analyzed by two-way ANOVA followed by Sidak’s multiple comparisons; n=6-11/group. Experimental details can be found in the Materials and Methods section.


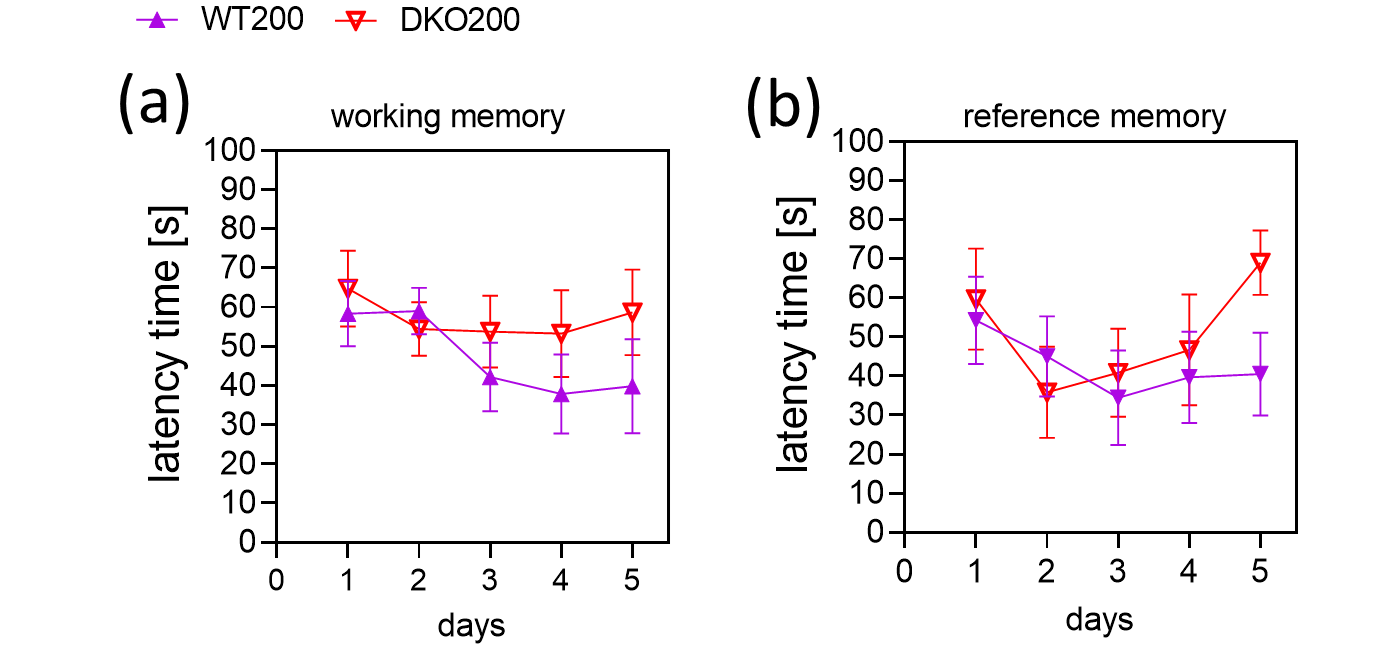


**Figure S12. Morris water maze performance test: working and reference memories.** The latency time increased with days in the DKO group pointing to a memory deterioration. Data are represented as means±S.E.M. Data were analyzed by two-way ANOVA followed by Sidak’s multiple comparisions; n=7-11/group. Experimental details can be found in the Materials and Methods section.


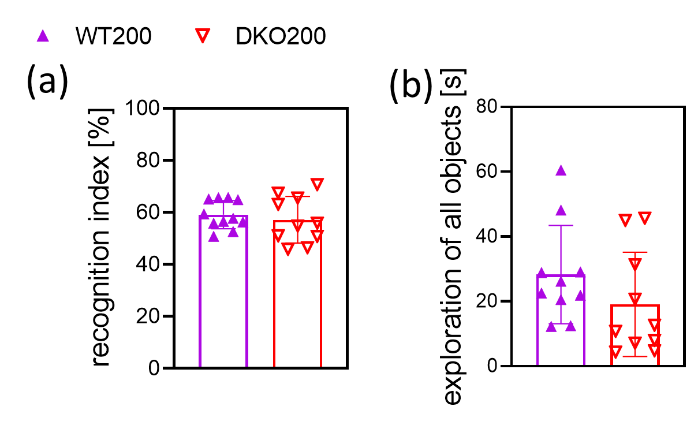


**Figure S13. Novel object recognition test: recognition index and exploration of all objects.** The duration of exploration of novel objects (expressed in seconds, s) tended to be shorter in the DKO group when compared to the WT group. Data are expressed as means±S.E.M. Data were analyzed by unpaired t-test; n=7-11/group. Experimental details can be found in the Materials and Methods section.


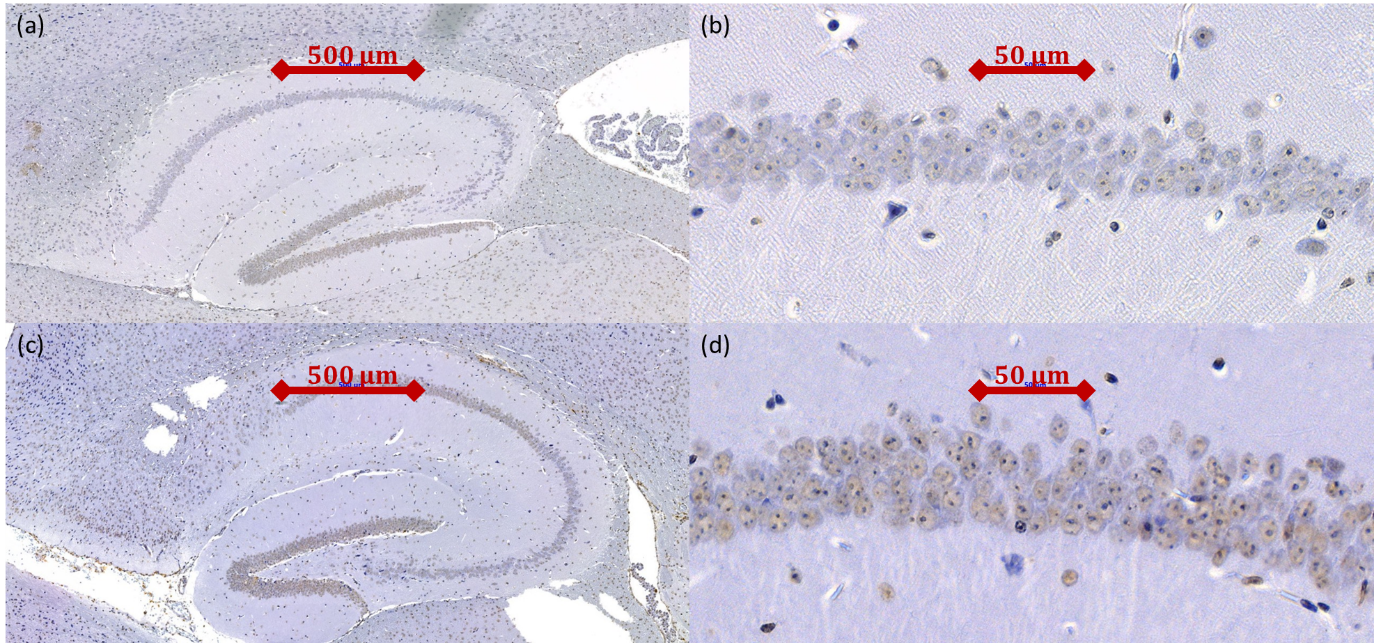


**Figure S14.** **TUNEL assay of the hippocampus in mice.** The WT (a,b) and DKO (c,d) mice were analyzed with 1x (a,c) and 40x (b,d) magnifications. The CA1 subfield of the hippocampus exhibited signs of neuronal apoptosis in the DKO mice (d), but no sign of cell death in the WT animals (c). Experimental details can be found in the Materials and Methods section.

**
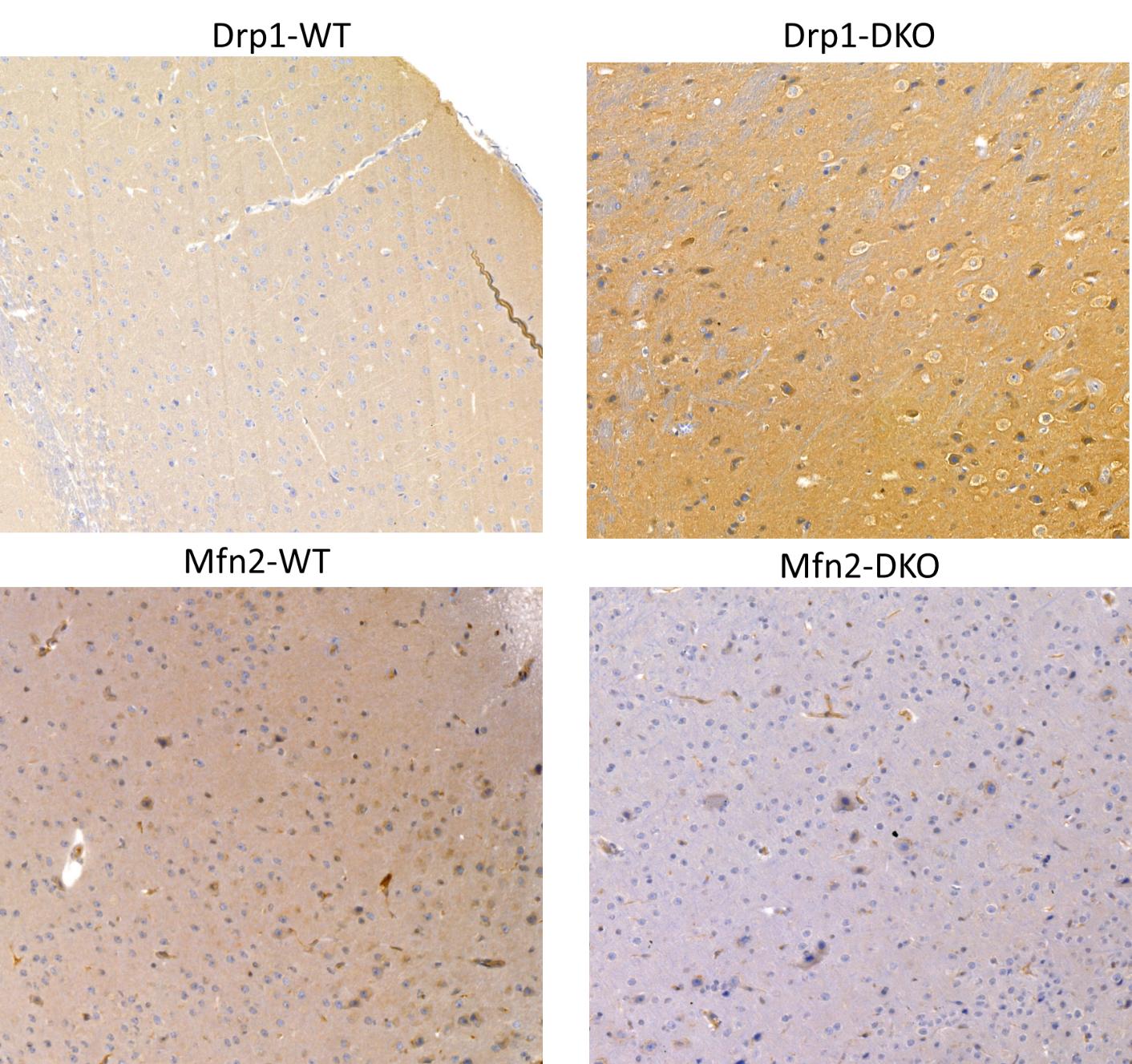
**

**
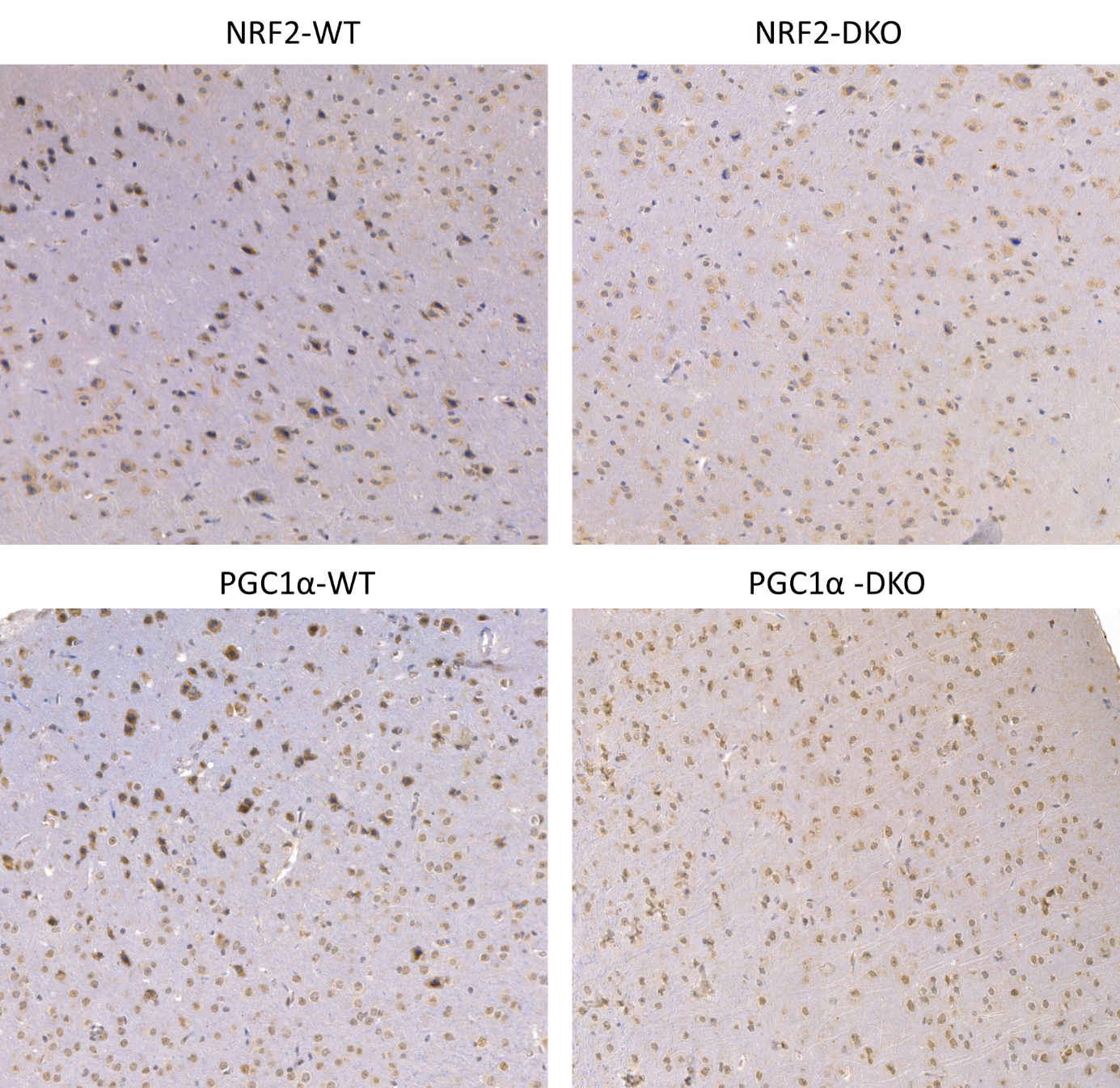
**

**Figure S15. Staining of the dynamin-related protein 1 (Drp1), mitofusin 2 (Mfn2), nuclear factor (erythroid-derived 2)-like 2 (Nrf2) and peroxisome proliferator-activated receptor gamma coactivator 1-alpha (PGC-1α) in the neuronal and glial cells of the WT and DKO mice.** Experimental details can be found in the Materials and Methods section.

**
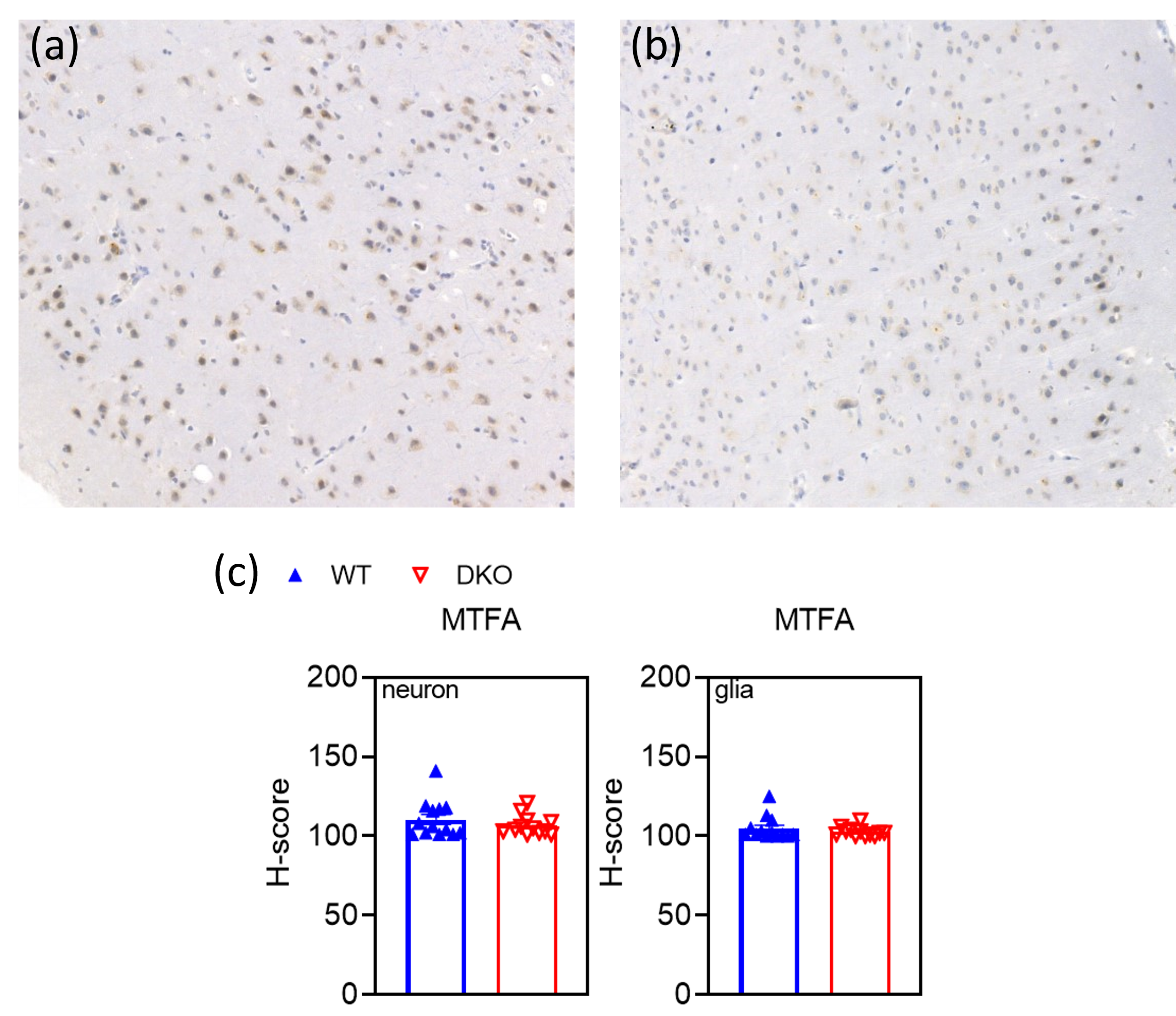
**

**Figure S16.** **Staining and expression level analysis of the** **mitochondrial transcription factor A (MTFA) in the neuronal and glial cells of the WT and DKO mice.** **A**: staining in the WT animal samples; **B**: staining in the DKO animal samples. The experimental data showed no significant differences using unpaired t-test. Data in panel **C** are expressed as means±S.E.M.; n=11-12/group. Experimental details can be found in the Materials and Methods section.

**
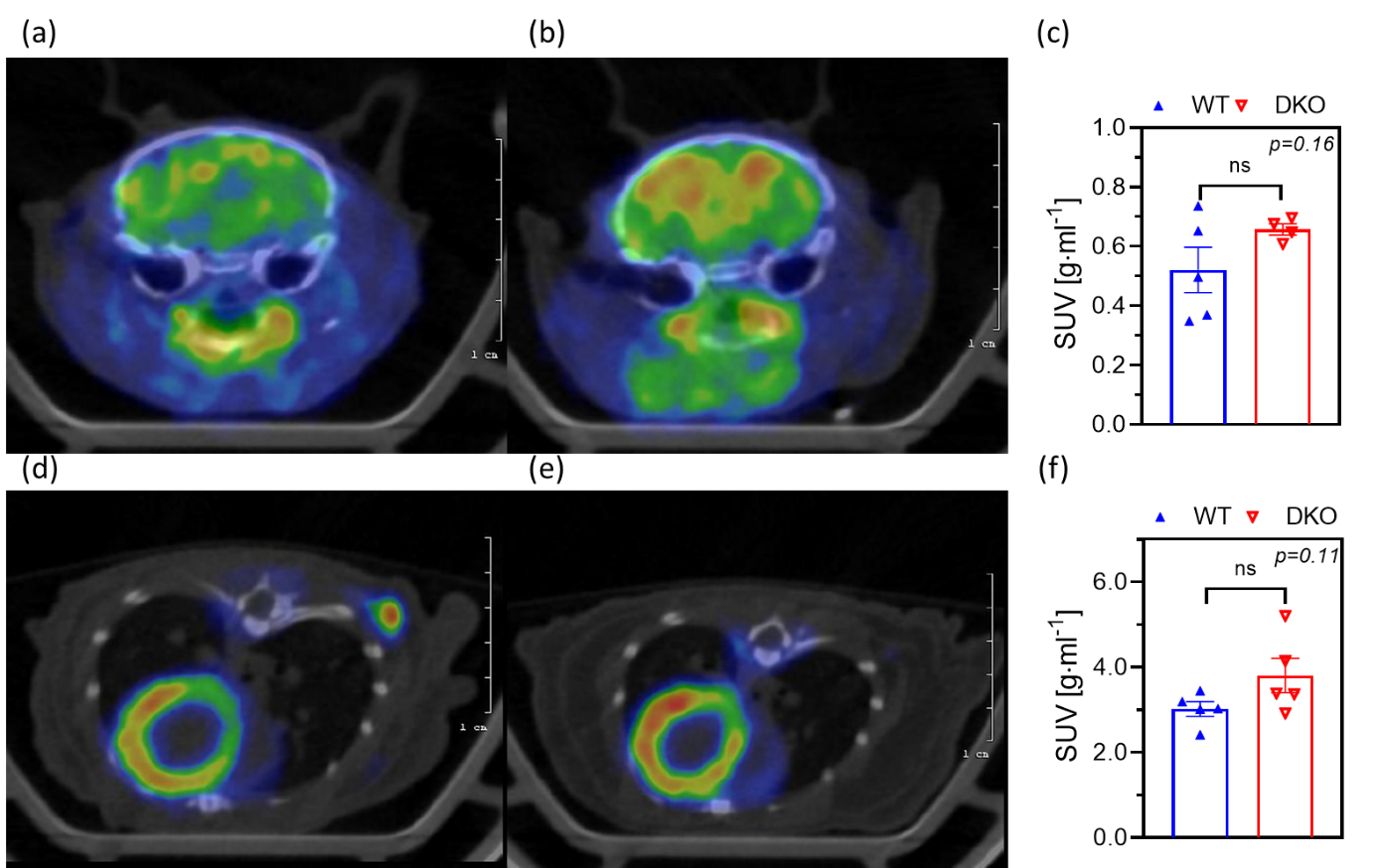
**

**Figure S17. ^18^F-Fluorodeoxyglucose uptake in PET-CT analysis. A**: WT brain**, B**: DKO brain, **D**: WT heart, **E**: DKO heart. Higher tendencies in glucose uptake were seen in the brain (c) and heart (f) tissues of the DKO mice relative to controls, pointing to either a compensatory higher glycolytic rate or inflammation caused by energy insufficiency. Data are expressed as means±S.E.M. Data were analyzed by unpaired t-test; n=4-5/group. Experimental details can be found in the Materials and Methods section.
